# Vitamin D as a central modulator of thyroid diseases: mechanisms and clinical implications

**DOI:** 10.3389/fimmu.2026.1748648

**Published:** 2026-01-26

**Authors:** Kewen Cheng, Yue Hu, Yuchuan Li, Yi Zhang, Junhui Wang, Chunhai Zhang

**Affiliations:** 1 Department of Thyroid Surgery, China-Japan Union Hospital of Jilin University, Jilin University, Changchun, China; 2 Department of Biobank, China-Japan Union Hospital of Jilin University, Changchun, China; 3 Lunenfeld-Tanenbaum Research Institute, Mount Sinai Hospital, Toronto, ON, Canada; 4 Thyropathy Hospital, Sunsimiao Hospital, Beijing University of Chinese Medicine, Tongchuan, Shanxi, China

**Keywords:** autoantibodies, dysbiosis, intestinal flora, thyroid autoimmune disease, thyroid cancer, vitamin D deficiency

## Abstract

Thyroid diseases are common endocrine disorders, with the incidence of thyroid cancer and autoimmune thyroid diseases rising worldwide. Vitamin D, a multifunctional steroid hormone, primarily regulates bone metabolism and calcium-phosphorus homeostasis. However, recent evidence increasingly supports the hypothesis that vitamin D plays a central role in the onset and progression of thyroid disorders, including both autoimmune and non-autoimmune conditions. In the present review, we summarize the correlation between vitamin D and thyroid disorders, elucidate the anticancer and immunoregulatory mechanisms of vitamin D in thyroid diseases, and explore its role in modulating gut microbiota. Additionally, we examine the applications in clinical settings of the use of vitamin D supplements in thyroid disorders, such as a preventive measure against cancer development and progression. Clarifying the mechanisms of vitamin D action in the development and progression of thyroid disease will support the design of interventional and early therapeutic strategies that not only prevent disease onset but also serve as a secondary chemopreventive approach to halt progression and enhance thyroid function.

## 1 Introduction

Thyroid diseases, as common disorders of the endocrine system, include autoimmune thyroid diseases (such as Hashimoto’s thyroiditis and Graves’ disease), thyroid dysfunction (hyperthyroidism, hypothyroidism), and thyroid cancers. Their pathogenesis involves the interaction of multiple factors, including genetics, environment, and immunity (1, 2). With the advancement of molecular medicine, the regulatory role of nutritional vitamins in the occurrence and progression of diseases has become a major research focus. In particular, the understanding of vitamin D’s functions has expanded beyond its traditional role as an essential micronutrient. It has now been confirmed to act in multiple capacities, such as a molecular cofactor, metabolic regulator, gene expression modulator, and epigenetic modifier. Through these diverse mechanisms, vitamin D helps coordinate the maintenance of cellular homeostasis and modulates key biochemical pathways involved in combating metabolic disorders, inflammatory responses, and immune regulation (3). Among the numerous vitamins, the research on vitamin D has attracted particular attention. It not only participates in calcium-phosphorus metabolism and bone health, but also plays important roles in immune regulation, cell proliferation, and differentiation. In recent years, the pathophysiological role of vitamin D in thyroid diseases has attracted widespread attention and the correlation between vitamin D deficiency and thyroid diseases is well established (4). Vitamin D not only participates in calcium-phosphorus metabolism and bone health but also plays important roles in immune regulation, cell proliferation, and differentiation. A large body of studies suggest that vitamin D may be involved in the development and progression of multiple thyroid diseases by inhibiting the proliferation and differentiation of thyroid cancer cells, modulating immune balance, suppressing autoantibody production, and affecting thyroid cell function (5–8). Additionally, epidemiological data indicate a higher prevalence of thyroid diseases in populations with vitamin D deficiency, though the exact mechanisms remain unclear (9). Furthermore, based on the concept of precision medicine, vitamin D has gradually evolved into an important tool in this field. It contributes to predicting, preventing, and delivering personalized treatments for chronic conditions such as cardiovascular diseases, diabetes, cancer, and neurodegenerative disorders. Among these advances, innovations in precision nutrition, including personalized vitamin D supplementation, have shown promise in improving treatment adherence and enhancing therapeutic outcomes (3). Therefore, this review aims to summarize the latest research progress on the association between vitamin D and thyroid diseases, explore the potential mechanisms of vitamin D deficiency in thyroid disorders, and integrate precision medicine technologies to provide a theoretical foundation for the clinical prevention and treatment of thyroid diseases.

## 2 Thyroid disorders

The thyroid gland is a vital endocrine organ located in the neck, just below the thyroid cartilage. Shaped like a butterfly, it is responsible for the production and release of thyroid hormones, thyroxine (T4) and triiodothyronine (T3), which play key roles in regulating metabolism, growth, development, and other physiological function (10). Thyroid disorders refer to a group of diseases characterized by abnormal thyroid function or structural changes. In the past few years, the prevalence of thyroid diseases has gone up substantially, evolving into a crucial global health problem (1). Thyroid disorders are broadly classified into the following categories: hyperthyroidism, hypothyroidism, thyroid autoimmune disorders, thyroid nodules, and thyroid cancers (11). Among them, the incidence of thyroid autoimmune disorders (e.g., Hashimoto’s thyroiditis, Graves’ disease) and thyroid cancer is considerably higher among women than among men, which may be related to estrogen levels and X-chromosome immunity genes (12, 13). This sex-related disparity in the occurrence of thyroid diseases further highlights the imperative need to enhance early screening and develop personalized treatment approaches to reduce the overall disease burden.

### 2.1 Thyroid cancer

Thyroid cancer (TC) is the ninth most common cancer in the world and can occur at all ages, especially in adolescents and adults under 40 years of age (14). There is a significant gender disparity in the incidence of thyroid cancer, with females accounting for approximately 75% of all thyroid cancer patients (15). In terms of origin, TC primarily originates from two types of thyroid parenchymal cells: follicular cells responsible for thyroid hormone synthesis and parafollicular C cells that produce calcitonin (16). Histologically, TC is classified into three main subtypes: 1) Differentiated TC (comprising over 90% of malignant thyroid tumors), such as papillary thyroid carcinoma (PTC) and follicular thyroid carcinoma (FTC); 2) Undifferentiated TC (relatively rare), encompassing poorly differentiated thyroid carcinoma (PDTC) and anaplastic thyroid carcinoma (ATC); 3) Special types (approximately 5% of TC cases), mainly medullary thyroid carcinoma (MTC) originating from parafollicular C cells (17).

Patients with thyroid cancer typically exhibit with no specific symptoms in the early stages. Clinically, most thyroid malignancies are diagnosed based on ultrasound characteristics and fine-needle aspiration cytology (14). Treatment usually involves surgical resection, and the 5 years survival rate following surgery usually is a high. Additionally, among the established risk factors including age, gender, race/ethnicity, and family history of TC, family history is the strongest predictor of disease risk. In addition to the above risk factors, Vitamin D deficiency has been shown to be strongly associated with thyroid cancer. A study lead by Zhao et al. indicated that vitamin D deficiency and decreased serum 25 - hydroxyvitamin D levels were related to an elevated risk of thyroid cancer compared with healthy populations (18).

### 2.2 Thyroid autoimmune disorders

Autoimmune thyroid diseases (AITDs), which is an autoimmune disorder specific to the thyroid gland, consists of chronic autoimmune thyroiditis, namely Hashimoto’s thyroiditis (HT), and Graves’ disease (GD) (2). AITDs are influenced by multiple factors, genetic, non-genetic, and environmental, and are considered familial disorders, with over 60% of cases exhibiting a positive family history. Among the non-genetic factors, Helicobacter pylori, hepatitis C virus (HCV), hantavirus, Toxoplasma gondii, human immunodeficiency virus (HIV), and alteration in intestinal microbiota are the most commonly implicated (19). Environmental risk factors associated with AITDs include smoking, excessive iodine intake, deficiencies in selenium and vitamin D, and certain occupational exposures (20). The pathology of AITDs is characterized by: (I) lymphocytic infiltration and the presence of thyroid autoantibodies in the circulation, and (II) the formation of lymphoid follicles with germinal centers and parenchymal atrophy, which represent the histological hallmarks (21).

Hashimoto’s thyroiditis (HT) is a prevalent autoimmune disorder affecting the thyroid gland, and it stands as the primary cause of hypothyroidism in areas worldwide where iodine levels are adequate. Incidence of AITDs is 7–10 times higher in women than in men (22). The disease is mainly characterized by destruction of thyroid cells due to lymphocytic infiltration, enhanced autoimmune response mediated by anti-thyroid peroxidase (TPOAb) antibodies and/or anti-thyroglobulin (TgAb) antibodies, and varying degrees of hypothyroidism, which usually requires lifelong treatment with levothyroxine (23).

Graves’ disease (GD) is one of the major AITDs and the most common cause of hyperthyroidism. It usually develops between the ages of 30 and 60, with women being 5–10 times more affected than men (12). The clinical hallmark of GD is thyrotoxicosis, resulting from breakdown of immune tolerance due to the binding of thyroid-stimulating hormone receptor (TSH-R) by the circulating antithyroid autoantibodies. This interaction stimulates the production and release of thyroid hormones, promotes thyroid cells proliferation, and lead to hypertrophy of the thyroid gland (24). Long-term disease management typically involves antithyroid medications such as propylthiouracil (PTU) and methimazole (MMI), radioactive iodine therapy, or thyroidectomy (25).

Currently, epidemiologic studies have shown that vitamin D influences the development of thyroid autoimmune diseases by modulating the body’s innate and adaptive immune mechanisms. Therefore, vitamin D deficiency is increasingly recognized as a key factor for the development of autoimmune thyroid diseases.

## 3 Vitamin D

Vitamin D is a multifunctional steroid hormone that is synthesized in the skin as a result of exposure to sunlight, and to a smaller degree, it can be obtained from the food we consume. In turn, skin and dietary vitamin D is converted to 25-hydroxyvitamin D [25(OH)D] or calcidiol by the liver enzymes 25-hydroxylase (CYP27A1 and CYP2R1). The inactive 25(OH)D is the primary circulating and storage form of vitamin D, which needs to be converted to the circulating biologically active compound 1,25-dihydroxyvitamin D (calcitriol) [1,25(OH)2D] by 1α-hydroxylase in the kidney to fulfill the physiological role of vitamin D (26, 27). Traditionally recognized for its crucial role in governing bone metabolism and maintaining calcium-phosphate balance, emerging proof indicates that vitamin D also exhibits extra-skeletal functions. These include modulating autoimmune disorders, cancer, metabolic syndrome, cardiovascular diseases, and diabetes (28, 29). Furthermore, 1, 25(OH)2D3 has been demonstrated to play significant roles in immune function across various cell types (e.g., lymphocytes, endothelial cells, osteoblasts, and keratinocytes), as well as in the control of cell growth and differentiation. Consequently, the pleiotropic functions of vitamin D include growth and bone calcification, immune system modulation, insulin secretion regulation, control of cellular proliferation, stimulation of cell differentiation, induction of apoptosis, safeguarding of calcium - phosphate stability, and regulation of muscular calcium transport (30, 31). For example, during the last few years, the relationship between vitamin D and thyroid diseases has received widespread attention, and several research findings have revealed that vitamin D inhibits the onset and development of thyroid cancer through multiple mechanisms: inhibiting tumor cell proliferation; triggering apoptosis; regulating cell cycle progression; inhibiting oncogenic signaling pathways (e.g., Wnt/β-catenin) (32). Furthermore, in the field of molecular genetics, studies have shown that the transcription factor VDR serves as the sole high-affinity target for 1,25(OH)_2_D. This finding indicates that the functional characteristics of VDR are almost identical to those of vitamin D. Moreover, vitamin D has been proven to exert an impact on the epigenome by regulating transcription factor binding, histone modification, chromatin remodeling, and three-dimensional chromatin organization, thereby altering the chromatin accessibility and transcription of genes associated with immunity, inflammation, cell apoptosis, and metabolism (33). In addition, it can also bind the vitamin D receptor (VDR) to modulate the immune cells, inhibit Th1 and Th17 cell differentiation, and promote Th2 and Treg cell activity, thus reducing the thyroid autoimmune response as well as affecting thyroid function.

## 4 Correlation between vitamin D and thyroid disorders

### 4.1 Correlation between vitamin D and thyroid cancer

**Figure 1:**
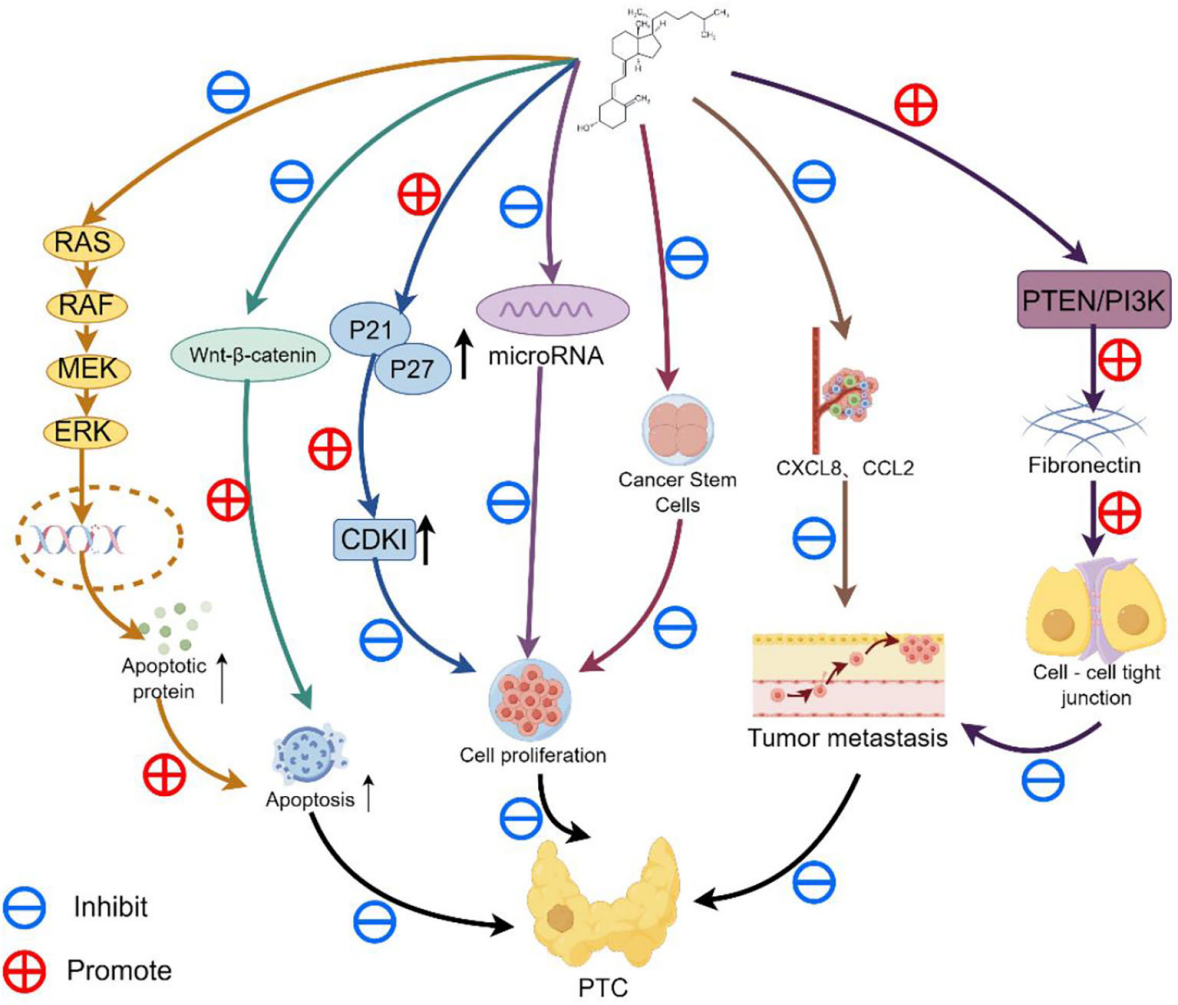
A schematic diagram demonstrates the anti-cancer mechanisms of vitamin D in thyroid carcinoma.

In recent years, accumulating experimental evidence demonstrates that vitamin D exerts anti-cancer effects by directly or indirectly inhibiting malignant tumor cell proliferation, invasion, and metastasis through binding to the VDR or via interactions with transcriptional regulators and cellular signaling pathways (9), as summarized in **Figure 1**.

#### 4.1.1 Cell cycle regulation

Vitamin D suppresses the expression of the proto-oncogene C-MYC while promoting the accumulation of the cell cycle inhibitory protein p27, thereby regulating the cell cycle and ultimately blocking abnormal proliferation of tumor cells. For example, the vitamin D receptor (VDR) directly binds to the C-MYC promoter and collaborates with DNA methyltransferases to form a repressive chromatin structure, thereby reducing C-MYC transcription. Additionally, vitamin D induces tumor-suppressive miRNAs such as miR-34a and let-7, accelerating the degradation of C-MYC mRNA. It can also downregulate deubiquitinating enzymes (e.g., USP28), thereby promoting proteasomal degradation of C-MYC to achieve profound suppression of C-MYC expression. Similarly, the vitamin D receptor directly binds to the p27 gene promoter to enhance its expression. Moreover, vitamin D inhibits the ubiquitin-mediated degradation of p27, thereby increasing its protein stability to arrest the cell cycle at the G1 and S phases (26, 34, 35).

#### 4.1.2 Epigenetic and stem cell regulation

As one of the core mechanisms of epigenomic regulation, chromatin remodeling affects gene accessibility by altering chromatin structure, thereby regulating the transcriptional process. It plays a critical role especially in the differentiation and therapeutic response of thyroid carcinoma. Studies have indicated that histone methyltransferase (SETMAR) can bind to the promoter region of the SMARCA2 gene, recruit histone acetyltransferases (HATs), and increase the level of histone acetylation in the promoter region, thereby activating the transcriptional expression of SMARCA2. As a core component of the chromatin remodeling complex, SMARCA2 can promote chromatin remodeling of differentiation-related genes (e.g., thyroglobulin Tg, thyroid peroxidase TPO, etc.), enhance the transcriptional accessibility of these differentiation-related genes, and thus induce the differentiation of thyroid carcinoma cells toward a mature phenotype (36). However, whether vitamin D-VDR can act as an exogenous regulatory factor to affect the differentiation of thyroid carcinoma by regulating histone modification, chromatin remodeling, and other processes has not been confirmed by relevant core studies, and further research is needed for further guidance. In addition, vitamin D can also epigenetically regulate microRNA (miRNA) expression, thereby interfering with the transcription of tumor-associated genes. For example, studies confirm vitamin D serves as a regulator of microRNA expression and cancer stem cell biology, and that it has an inhibitory effect on the growth and proliferation of cancer cells. The underlying mechanisms include vitamin D–mediated upregulation of tumor-suppressive microRNAs (e.g., miR-22, miR-34a, and let-7) and downregulation of oncogenic microRNAs (e.g., miR-21 and miR-155), thereby enhancing the expression of tumor suppressor genes (such as PTEN) and pro-apoptotic factors to inhibit pro-tumorigenic signaling pathways, and blocking the growth and proliferation of cancer cells at the gene transcription level (7, 37). Additionally, vitamin D can also target and inhibit cancer stem cells, attenuating the stemness characteristics of tumor cells. For instance, the vitamin D-VDR signaling pathway can exert an anti-tumor effect by regulating the expression of stemness-associated genes such as Oct4, Nanog, and Sox2 (38), and interfering with signaling pathways including Wnt/β-catenin, AKT/ERK, Notch, and Hedgehog to target and inhibit the proliferation of cancer stem cells (39).

#### 4.1.3 Metastasis suppression

Vitamin D-activated VDR can alter the chromatin status of epithelial-mesenchymal transition (EMT)-related genes by recruiting chromatin remodeling complexes. For example, on the one hand, it inhibits the expression of EMT-inducing factors such as Snail, Slug, and Twist (36); on the other hand, it promotes the expression of EMT-suppressing factors such as E-cadherin and ZO-1, maintaining epithelial cell polarity and intercellular junctions (40). *In vitro* studies have confirmed that vitamin D treatment can significantly inhibit the expression of Snail, induce the expression of E-cadherin, and reduce cell migration and invasion in the human follicular thyroid carcinoma (FTC) cell line FTC-133 (41, 42). Furthermore, through the PTEN/PI3 kinase pathway, vitamin D enhances fibronectin expression and reverses the cadherin switch (epithelial-mesenchymal transition, EMT), strengthening intercellular junctions and consequently reducing cancer cell invasion and metastasis. As a key tumor suppressor, PTEN antagonizes the lipid kinase activity of PI3K, thereby suppressing AKT expression. The downregulation of AKT leads to reduced expression of EMT transcription factors, which in turn inhibits mesenchymal marker expression while promoting epithelial marker expression. This strengthens epithelial cell adhesion and stabilizes the basement membrane structure, ultimately suppressing cancer cell invasion and metastasis (43, 44).

#### 4.1.4 Anti-tumor inflammation modulation

Vitamin D has also been shown to inhibit pro-tumor inflammation by reducing the inflammation mediators such as cytokines TNF-α and IL-6. Studies have found that NF-κB is a core signaling pathway for pro-inflammatory factors, promoting the expression of cytokines like TNF-α and IL-6. Vitamin D can inhibit IκB kinase (IKK), prevent the degradation of IκB, and thereby downregulate NF-κB levels, reducing the expression of pro-inflammatory factors. Additionally, research indicates that vitamin D can suppress JAK1/STAT3 phosphorylation, diminishing IL-6-mediated signaling pathways and consequently blocking pro-tumor inflammatory responses. Furthermore, it can downregulate the activity of p38 and JNK, influencing the MAPK signaling pathway to reduce TNF-α-induced inflammatory responses, thereby achieving an anti-tumor effect (45, 46).

All the above mechanisms demonstrate that elevated serum vitamin D concentration exerts an inhibitory effect on the occurrence and development of thyroid cancer, and previous studies have indicated that this inhibitory effect is closely associated with the expression of VDR. Numerous studies have indicated that compared with normal thyroid tissues and benign thyroid diseases, the expression levels of VDR protein and mRNA are decreased in papillary thyroid carcinoma (PTC). A basic study conducted by Pang et al., which investigated the functional effects of VDR knockdown on PTC from three aspects—cell proliferation, apoptosis, and invasion—found that in vitamin D-treated cells, compared with the control group, the mRNA expression levels of Wnt3 and CTNNB1 in the VDR knockdown group were significantly increased. This indicated that vitamin D inhibits this pathway via VDR, whereas VDR knockdown activates the pathway (47). This study clarified the key mediating role of VDR in vitamin D-regulated PTC progression; however, it still has limitations. For instance, it only explored the role of VDR knockdown in vitamin D-treated PTC cells, so numerous additional experiments are required to improve and validate these findings. However, a study conducted by Zhang et al. drew an opposite conclusion. An analysis of VDR and vitamin D levels in 156 patients with thyroid diseases revealed that VDR was highly expressed in PTC tissues (65.4%), and vitamin D induced apoptosis of PTC cell lines in a dose-dependent manner (48). The reason why the VDR expression level is inconsistent with the conclusions mentioned above may be related to the single source of samples, small sample size and the lack of animal experiment verification in this study. Furthermore, clinical studies have revealed that decreased VDR protein and mRNA expression in PTC is related to low serum vitamin D levels (49). In contrast, reduction of 25(OH)D levels in serum might elevate the likelihood of developing thyroid cancer, leading to the conclusion that vitamin D deficiency as well as low expression of VDR in human cells may represent risk factors for the development of thyroid cancer. For example, a number of clinical investigations have shown that pharmacological doses of 1,25(OH)_2_D or its analogs can suppress thyroid cancer growth in patients (26). In the context of gene expression, vitamin D inhibits the proliferation of thyroid cancer cells by suppressing the expression activity of gene pathways involved in cell growth and survival. For example, 1,25-(OH)2D3 can inhibit the activity of papillary thyroid cancer cell line TPC-1 and promote apoptosis of cancer cells, potentially through suppression of the Ras-MEK-ERK signaling pathway. This inhibition may, in turn, reduce cell proliferation and modulate the expression of apoptosis-related proteins (50). Additionally, vitamin D can influence the risk and progression of thyroid cancer by modulating gene transcription. For example, vitamin D regulates the transcription of target genes by binding to the VDR (51). This receptor binds to vitamin D response elements (VDREs) on target genes through its DNA-binding domain, thereby exerting transcriptional regulatory effects (52). Furthermore, after binding to intracellular VDR, 1,25-(OH)2D3 induces conformational changes in VDR, which then forms a heterodimer complex with the retinoid X receptor (RXR). This complex subsequently regulates the transcription of downstream genes, thereby modulating cell proliferation, differentiation, apoptosis, and immune function (53). In the field of nutrigenomics, Single nucleotide polymorphisms (SNPs), as the most widespread form of genetic variation in the genome, can fine-tune the structure and function of gene-encoded products. This subsequently influences an individual’s metabolic efficiency of vitamins and signaling activity, thereby determining susceptibility to thyroid cancer and shaping the characteristics of disease progression. For example, the authoritative review (54) notes that VDR, as the core transcription factor mediating the biological functions of vitamin D, harbors multiple functional SNP loci in its gene. Among these, FokI (rs2228570), TaqI (rs731236), and BsmI (rs1544410) are the most extensively studied sites (55). Variants at these SNP loci can alter receptor activity, thereby affecting the efficiency of vitamin D signaling, and are closely associated with the prognosis of thyroid cancer. A study focusing on six vitamin D - related genes (VDR, CYP2R1, CYP24A1, CYP27B1, DHCR7, and CUBN) demonstrated that the regulatory functions of vitamin D and its associated genetic pathways are influenced by their metabolic enzymes, thereby exerting potential roles in inhibiting the occurrence and progression of thyroid cancer (56). For example, in the vitamin D metabolic pathway, SNP variations in 1α-hydroxylase (CYP27B1) and 24-hydroxylase (CYP24A1) directly regulate the activation and inactivation efficiency of vitamin D, forming key nodes of gene-vitamin interactions, thereby affecting tumor progression. For instance, CYP27B1 can promote vitamin D activation and inhibit the proliferation of thyroid carcinoma stem cells; whereas CYP24A1, by inactivating vitamin D, attenuates its anti-tumor effects and promotes tumor progression (57). Furthermore, further studies have indicated that the transcriptional regulatory function of vitamin D is also affected by genetic polymorphisms. For example, genetic studies have demonstrated that polymorphisms in VDR gene SNP loci (e.g., FokI, TaqI, BsmI, etc.) are closely associated with the invasiveness of thyroid cancer. Patients carrying the FokI FF genotype are more prone to developing thyroid cancer with higher malignancy and lymph node metastasis (58), while the TaqI TT genotype is correlated with larger tumor volume, distant metastasis, and poorer survival rates (59). These variations may exert effects by altering the ligand-binding affinity, transcriptional activity, or post-translational modification status of VDR, thereby regulating the vitamin D signaling pathway. From an immunological perspective, CXCL8 and CCL2 are two chemokines secreted by thyroid tumor cells, and these chemokines exert a variety of pro-tumorigenic effects, including increased metastatic potential. Recent studies have demonstrated that vitamin D treatment significantly reduces metastasis potential of thyroid cancer cells. This effect is attributed, in part, to vitamin D’s ability to inhibit the secretion of CCL2 in various thyroid cancer cell lines and CXCL8 specifically in TPC-1 cells (60).

Meanwhile, the active form of vitamin D (calcitriol) also exerts its anti-cancer effects by binding to VDR. Several studies have elucidated the anti-tumor mechanisms of calcitriol. For instance, calcitriol upregulates the expression of cyclin-dependent kinase inhibitors (CDKIs), which has a significant inhibitory effect on cell proliferation (7). Additionally, calcitriol can negatively regulate cell growth and proliferation by influencing microRNA expression (46). Furthermore, calcitriol promotes tumor cell apoptosis by inducing the expression of cysteine-aspartic proteases (caspases) alongside other pro-apoptotic proteins (BAX, BAK, and BAD) (8). In summary, vitamin D levels are closely related to the occurrence and development of thyroid cancer, and this correlation may be achieved through VDR-mediated epigenomic and transcriptional regulation. The main core mechanisms include inhibiting cell proliferation, inducing cancer cell apoptosis, and suppressing cancer cell invasion. Therefore, vitamin D supplements may serve as a preventive strategy for thyroid cancer in the future, which can not only inhibit the occurrence of the disease but also suppress its progression.

### 4.2 Correlation between vitamin D and thyroid autoimmune disorders

The importance of vitamin D in biological functions is emphasized by the fact that VDRs are present in various tissues of the body and have expression in numerous cells, such as muscle cells, intestinal epithelial cells, kidney cells, and immune cells (61). The widespread expression of VDR in a variety of immune cells, including monocytes/macrophages, dendritic cells, as well as B and T cells (62) suggests vitamin D plays some key roles in the regulation of the innate and adaptive immune systems. Vitamin D has been found to play a crucial role as a mediator in the innate immune response. For instance, vitamin D promotes the transformation of monocytes into macrophages, consequently enhancing the chemotactic ability and phagocytic activity of innate immune cells. In contrast, in adaptive immunity, vitamin D promotes autoantigen tolerance by inhibiting the differentiation and maturation of dendritic cells (DCs) in lymph nodes and preserving the immature phenotype (63). It suppresses T-cell proliferation, including the proliferation of Th1 cells and the production of cytokines (IL-2 and interferon-γ) as well as Th17-derived cytokines (IL-17 and IL-21), while promoting the production of anti-inflammatory Th2 cytokines (IL-3, IL-4, IL-5, and IL-10). This leads to a shift from Th1 and Th17 phenotypes toward a Th2 phenotype (64–66). Additionally, in terms of immune regulation, vitamin D promotes the development of regulatory T cells (Tregs) by influencing DCs and directly targeting T cells, thereby blocking Th1 differentiation (5, 67). Specifically, vitamin D can inhibit the activity of pro-inflammatory immune cells (Th1 and Th17 cells) via the VDR/RXR signaling pathway, promote the generation of regulatory T cells (Tregs), and reduce the production of thyroid-targeting autoantibodies (e.g., TPO-Ab and TG-Ab), thereby maintaining immune tolerance of the immune system to thyroid tissue (68–70). Meanwhile, in terms of B-lymphocyte regulation, vitamin D can affect B-cell homeostasis by inhibiting follicular helper T cells and thereby affecting B-cell homeostasis. For instance, it suppresses the activation and proliferation of naïve B cells, triggers B cell programmed cell death, and hinders the transformation of B cells into plasma cells as well as the synthesis of immunoglobulins (71).

**Figure 2:**
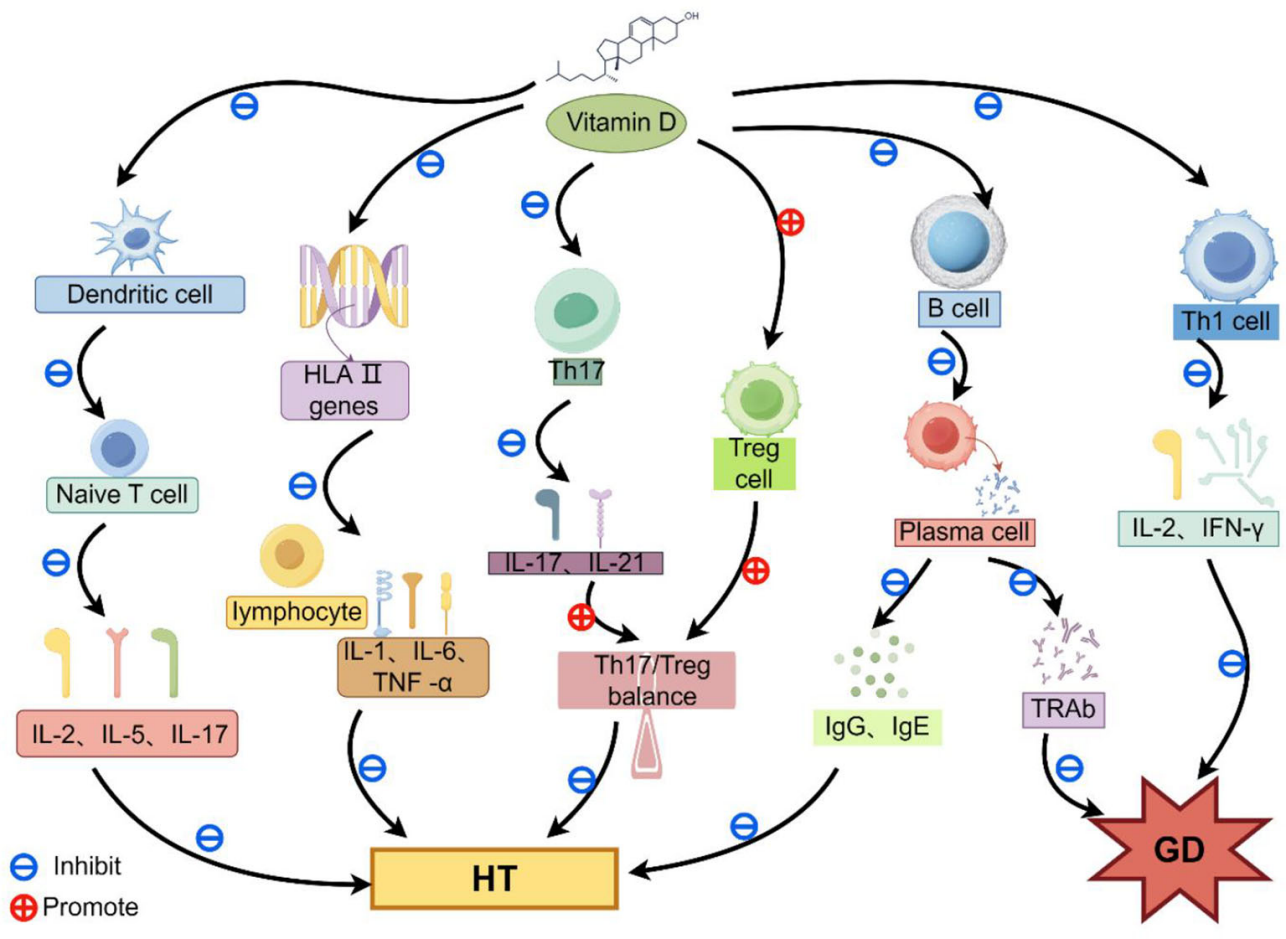
A schematic diagram showing the immunosuppressive effect of vitamin D in autoimmune thyroid diseases. Vitamin D exerts an immunosuppressive effect on Hashimoto's thyroiditis (HT) by preventing dendritic cell (DC)-dependent T cell activation, downregulating the expression level of HLA class II genes in the thyroid, inhibiting B cell activity, and restoring the Th17/Treg balance.

Therefore, a deficiency in vitamin D can damage the integrity of the immune system, leading to inappropriate immune responses and thereby contributing to the development of autoimmune thyroid diseases (19, 67, 72, 73). Current research suggests that vitamin D may regulate various immune mechanisms to control autoimmunity and improve thyroid function, as illustrated in **Figure 2**. For example, vitamin D been proposed to contribute to the suppression of the immune process of HT (74). This study described several aspects of vitamin D inhibition on HT immune responses: I. Vitamin D attaches to the VDR located on the surface of DCs and inhibits DC - mediated T - cell activation. This suppression results in a decrease in the synthesis of pro - inflammatory cytokines, including interleukin - 2 (IL - 2), interleukin - 5 (IL - 5), and interleukin - 17 (IL - 17), and diminishes the cytokine - driven immune reactions (75). II. Vitamin D suppresses lymphocyte proliferation, differentiation, and the secretion of pro-inflammatory cytokines by downregulating the expression of HLA class II genes in the thyroid, thereby inhibiting immune-inflammatory reactions. III. Vitamin D can reduce the proliferation and differentiation of B cells into plasma cells, thereby suppressing the excessive secretion of immunoglobulins such as IgG and IgE (76), ultimately mitigating damage to thyroid cells. VI. Vitamin D inhibits the proliferation and differentiation of Th17 cells, and reduces the release of pro-inflammatory cytokines (IL-17 and IL-21) which are derived from Th17 cells. Simultaneously, vitamin D promotes the differentiation of Treg cells, and restores the balance of the Th17/Treg cell ratio, thereby decreasing the occurrence of Th17 cell-induced inflammation of the thyroid gland (77).

Immunosuppressive Effects of Vitamin D on Graves’ Disease (GD) through multiple mechanisms, including the suppressing B cells and autoantibody production, as well as the inhibiting pro-inflammatory cytokine secretion. Similarly, vitamin D also suppresses the onset and progression of GD by modulating T and B lymphocytes and regulating their immune response. For instance, Th lymphocytes can be divided into two subsets: Th1 and Th2 (78). Th1 lymphocytes secrete IFN-γ and IL-2, which are closely associated with cell-mediated immune responses, whereas Th2 lymphocytes produce IL-4 and IL-5, which are linked to humoral immune responses (79). Among these, Th1-mediated cellular immunity plays a predominant role in the immunopathogenesis of GD (80). Therefore, vitamin D can mitigate autoimmune-mediated damage to the thyroid gland by inhibiting Th1 cell differentiation and regulating the secretion of cytokines such as IFN-γ and IL-2 (81, 82). Additionally, GD is directly associated with B lymphocytes, as its specific autoantibody, TRAb, is produced by B cells during autoimmune response. Thus, in the immune system, vitamin D may reduce the production of TRAb by inhibiting the proliferation and differentiation of B cells into plasma cells, thereby alleviating thyroid gland irritation and damage (71). In summary, vitamin D may attenuate the evolution of GD by downregulating Th1 and B lymphocytes.

### 4.3 The impact of vitamin D on thyroid disorders through gut microbiota modulation

**Figure 3:**
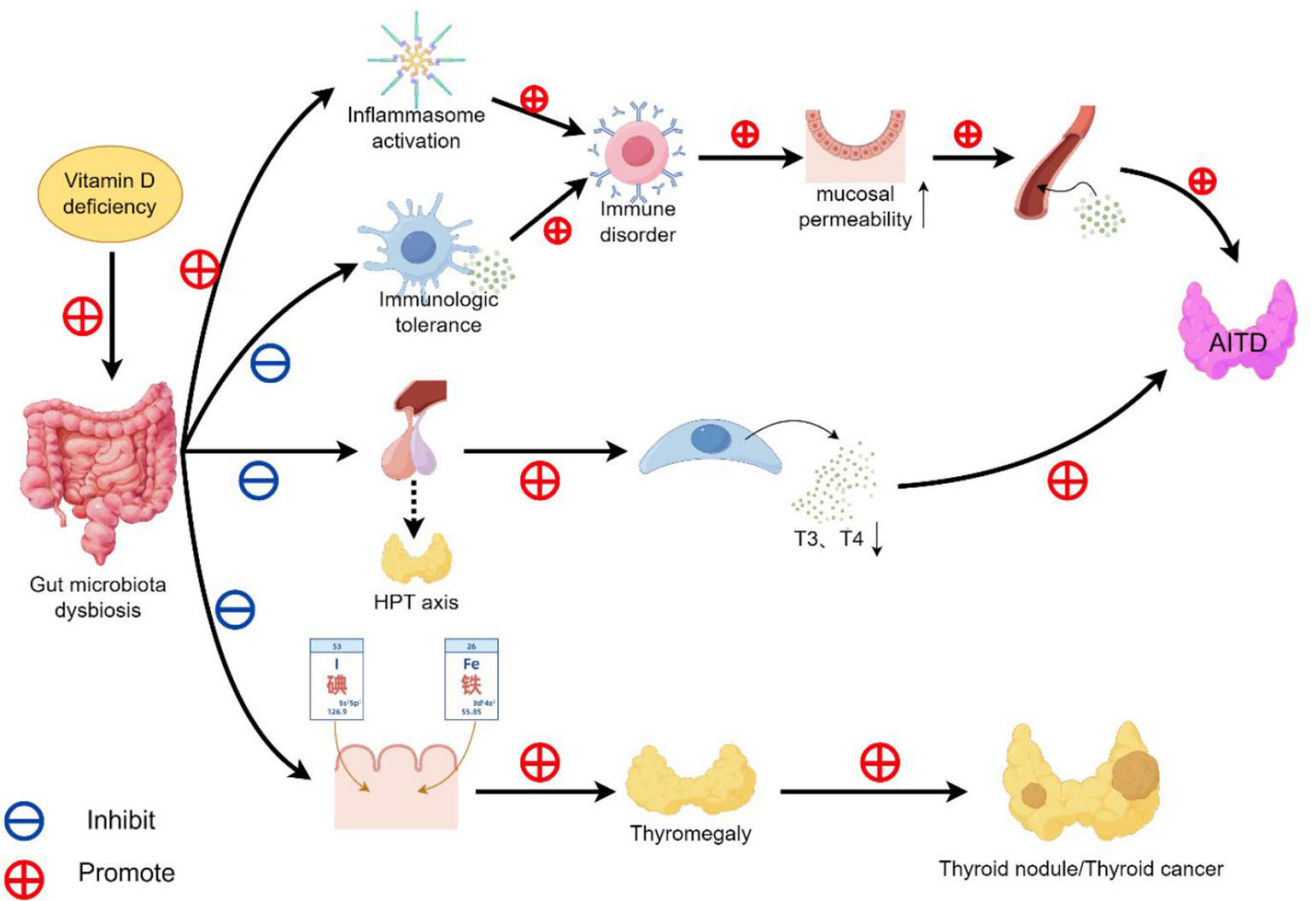
Schematic illustration showing the effects of vitamin D on the thyroid-endocrine-gut axis.

To investigate the relationship between vitamin D and thyroid diseases, some groups have explored the interactions among vitamin D, gut microbiota, and thyroid diseases from the perspective of the gut-endocrine-thyroid axis, as illustrated in **Figure 3**. Gut bacteria play a role in the synthesis of vitamins (vitamin K, folate, vitamin B, and vitamin D) (83, 84), the digestion of dietary fiber, and the regulation of immune responses (85). Additionally, research has found that gut microbiota can determine the function of the immune system (86). For example, a meta-analysis examining the association between autoimmune thyroid disease (AITD) and the gut microbiota included eight studies comprising a total of 196 AITD patients and 160 healthy controls. The results indicated that the abundance of beneficial bacteria was significantly reduced, whereas the abundance of harmful bacteria and certain commensal bacteria was increased. Moreover, specific microbial taxa were significantly correlated with thyroid autoantibodies, including TPOAb and TRAb. The overgrowth of certain bacteria caused by dysbiosis disrupts immune homeostasis by promoting excessive activation of inflammasomes and reducing immune tolerance. It also damages the intestinal mucosa, increases intestinal permeability, and reintroduces antigens, thereby triggering local inflammation and promoting the occurrence of AITD (87). However, this study has certain limitations, such as a small number of included studies and a lack of support from multi-center and large-sample data. In addition to affecting the immune system, gut microbiota also plays a critical role in the progression of thyroid diseases by regulating thyroid function through the absorption of thyroid-related micronutrients (88). For instance, reduced richness and diversity of gut microbiota can impair the uptake of thyroid-related minerals (such as iodine and iron). A common consequence of this impairment is poor iodine absorption, which may lead to goiter, thyroid nodules, or even thyroid cancer (89). Similarly, iron deficiency may disrupt thyroid hormone synthesis, storage, and secretion, which increases thyrotropin (TSH) secretion and causes thyroid enlargement (90, 91). Moreover, microbiota can influence TSH secretion via the hypothalamic-pituitary axis (92). For example, Zhao et al. conducted a gut microbiota study focusing on hypothyroidism patients. Using a two-cohort design involving discovery and validation phases, they performed 16S rRNA gene sequencing to characterize the gut microbiota in 50 patients with HT and 27 healthy controls. The results demonstrated that HT patients presented with significant gut microbiota dysbiosis, and the gut microbial composition was closely correlated with core clinical parameters of HT, including TPO-Ab and TG-Ab. Similarly, Ishak et al. conducted qualitative and quantitative analyses of the gut microbiota in 29 patients with HT and 12 healthy controls. Their findings indicated that HT patients exhibited marked gut microbiota dysbiosis, and such alterations in microbial composition might contribute to the pathogenesis of HT via mechanisms including immune modulation and intestinal barrier disruption (93, 94). Despite the relatively small sample sizes in the two aforementioned studies, both adopted a study design incorporating discovery and validation cohorts and yielded consistent conclusions.

Furthermore, Yu et al. characterized the gut microbiota in 90 thyroid carcinoma (TC) patients and 90 healthy controls (HC) using 16S rRNA gene sequencing. Their analyses revealed that TC patients exhibited decreased gut microbial abundance and diversity, along with reduced levels of short-chain fatty acid (SCFA)-producing bacteria, such as Trichosporon and Butyricicoccus (95). In an animal study conducted by Ooi et al., vitamin D was found to maintain intestinal homeostasis by regulating the composition of gut microbiota. Vitamin D deficiency, by contrast, induces gut dysbiosis and increases susceptibility to colitis (96). Similarly, a systematic review has confirmed the association between vitamin D deficiency and gut dysbiosis from both animal and human experimental perspectives. Specifically, one of the included interventional studies demonstrated that vitamin D supplementation can alter the composition of gut microbiota and promote an increase in the abundance of beneficial bacteria (97). Collectively, these lines of evidences suggest that vitamin D may modulate the composition of gut microbiota by maintaining intestinal homeostasis, thereby enriching beneficial bacteria (e.g., enhancing the diversity and abundance of gut microbiota) and reducing the risk of thyroid cancer. Consequently, vitamin D deficiency could lead to dysbiosis of the intestinal flora, which may inhibit the sodium-iodide symporter (NIS), impairing thyroidal iodine uptake and weakening immune function. This disruption may contribute to thyroid dysfunction, such as AITD and thyroid cancer (98). Additionally, vitamin D helps maintain the integrity of intestinal tight junctions (TJ) (99). For example, Kong et al. demonstrated an increase in intestinal mucosal damage in animal model mice after knocking out the vitamin D receptor gene, suggesting a protective effect of vitamin D on the intestinal mucosa (100). Whereas the disruption of tight junctions (TJs) causes pathogens to translocate to neighboring mesenteric lymph nodes and promotes inflammation and autoimmunity, the main cytokines involved in the inflammatory process are TNF-α and INF-γ, which may also be relevant factors involved in the pathogenesis of thyroid autoimmune disorders, such as HT, GD, and others (101–103). In summary, a robust thyroid-endocrine-gut axis exists in the human body, where gut microbiota is strongly correlated with thyroid function. Moreover, the homeostasis of gut microbiota has been confirmed to be regulated by vitamin D, which can reduce the incidence of autoimmune thyroid diseases and thyroid cancer through multiple mechanisms such as increasing the abundance of beneficial microbiota and protecting the mucosal barrier.

## 5 Clinical applications of vitamin D supplementation in thyroid disorders

The influence of vitamin D on the development and progression of thyroid cancer is well documented. A clinical study involving 235 patients with papillary thyroid carcinoma (PTC) and 108 healthy controls demonstrated that, compared with the healthy control group, 166 out of the 235 thyroid carcinoma patients had significantly decreased serum levels of 25-hydroxyvitamin D (104). Furthermore, in another clinical study enrolling 548 patients with PTC, preoperative serum levels of 25-hydroxyvitamin D were also found to be significantly lower in patients with tumor diameter >1 cm and/or tumor metastasis (105). Correspondingly, a prospective cohort study revealed significantly lower serum levels of 25-hydroxyvitamin D in patients with PTC compared with healthy controls, based on a direct comparison of serum vitamin D concentrations between the two groups (106). In contrast, in a study involving 100 patients who underwent total thyroidectomy found that patients with vitamin D deficiency during the perioperative period were more likely to have thyroid malignancies in their postoperative histopathological evaluations (107). Moreover, numerous studies have also demonstrated low levels of serum 25(OH)D, as well as a reduction in its conversion to 1,25(OH)2D3 function, increase the risk of thyroid malignancy (108). However, the results across studies remain highly controversial. Some studies have reported no significant difference or no association in serum 25-hydroxyvitamin D levels when comparing experimental and control groups (108–111). But, these studies are not without limitations—for example, marked age disparities between the control and case groups, as well as an uneven distribution of disease subtypes—which may render their results susceptible to confounding bias. Thus, based on aforementioned research, vitamin D deficiency and decreased circulating osteotriol are associated with the development and progression of thyroid cancer (**Table 1**). Therefore, it appears reasonable to consider vitamin D supplementation as a cancer preventive strategy, both for reducing the risk of disease onset and as a secondary chemopreventive measure to inhibit disease progression (112). From the perspective of nutrigenomics, vitamin D acts as a key epigenetic regulator that mediates nutriment-gene interactions by modulating the epigenetic status of thyroid cancer-related genes, thereby achieving dynamic regulation of tumorigenesis and progression (113, 114). For instance, accumulating evidence indicate (115, 116) that DNA methylation represents a crucial epigenetic mechanism through which vitamin D modulates the expression of thyroid cancer-associated genes. Chronic vitamin D deficiency can upregulate the activity of DNA methyltransferases (DNMTs), thereby inducing hypermethylation in the promoter regions of VDR target genes (e.g., E-cadherin). This hypermethylation event suppresses gene transcription, ultimately leading to the undifferentiated state of tumor cells with potent oncogenic potential. Conversely, adequate vitamin D intake inhibits DNMTs activity, reduces the methylation levels of tumor suppressor genes, restores their transcriptional expression, and facilitates cellular differentiation. These findings highlight the critical role of early vitamin D intervention in the prevention of thyroid cancer. For example, according to a recent meta-analysis, it suggests that increasing serum 25(OH)D concentrations through supplementation may help reduce cancer-related mortality (117). Consistent with these findings, a zoological study (118) also demonstrated that vitamin D supplementation reduced tumor growth rate, diminished tumor size, and inhibited tumor progression. In this mouse model study, the animals were divided into two groups: one group received vitamin D supplementation, while the other did not. The results showed that tumor growth was significantly slower in the vitamin D-supplemented group compared with the control group. The underlying mechanism is thought to involve vitamin D supplementation regulating cellular signaling pathways, promoting apoptosis, and inhibiting angiogenesis—effects that restrict the blood supply to tumors, thereby suppressing cancer cell growth and gradually reducing tumor volume. However, the efficacy of vitamin D supplementation is contingent upon dosage and treatment timing. To translate these preclinical findings into cancer prevention and treatment strategies for humans, extensive further research and well-designed clinical trials are required to validate its potential and safety (119, 120). Therefore, when determining the optimal clinical dosage of vitamin D for the chemoprevention of thyroid cancer, it is imperative to incorporate nutrigenomics-based precision medicine approaches and tailor therapeutic regimens according to individual patient responses. Precision medicine aims to customize prevention and treatment strategies based on individual variability in genetics, environment, and lifestyle. Its integration with vitamin D nutritional interventions is reflected in etiology-specific interventions and biomarker-guided personalized therapies (121). However, extensive observational studies and controlled clinical trials are required to evaluate the optimal individualized anticancer efficacy of vitamin D analogs across distinct etiological mechanisms and specific biomarkers. A study focusing on vitamin D-mediated interventions for disease prevention and treatment under the framework of precision medicine emphasized the need for individualized therapeutic regimens tailored to the specific etiologies or pathophysiological pathways of different cancer types (119). For instance, in prostate cancer, vitamin D deficiency promotes β-catenin-mediated epithelial-mesenchymal transition (EMT), thereby enhancing tumor invasiveness. To target this specific pathogenic mechanism, high-dose vitamin D_3_ (40,000 IU/day) can inhibit the Wnt/β-catenin pathway and reduce tumor invasiveness, but this therapeutic effect is only observed in patients with the wild-type VDR gene. In contrast, carriers of the VDR FokI polymorphism require combined treatment with CYP24A1 inhibitors to prevent the metabolic inactivation of vitamin D. This underscores the intervention logic of integrating etiological mechanisms with genotypic profiles (122, 123). Additionally, in colorectal cancer, targeting the etiology of inflammation-driven intestinal epithelial carcinogenesis, vitamin D supplementation exerts a risk-reduction effect in Asian populations by inhibiting the NF-κB pathway. Notably, this preventive effect is more pronounced in proximal colon cancer, where inflammation-driven characteristics are more prominent, than in distal colon cancer, which exemplifies the specificity of interventions based on both etiological mechanisms and lesion locations (119). However, whether these pathogenic mechanisms are also operative in thyroid cancer remains an open research question. Secondly, for biomarker-guided personalized dosing strategies, VDR expression levels and serum 25(OH)D serve as core predictive biomarkers for treatment efficacy in the field of cancer. For instance, in oral cancer patients with high VDR expression, calcitriol supplementation reduces the risk of tumor recurrence. In contrast, those with low VDR expression require combination with chemotherapy to enhance treatment efficacy (124). In colorectal cancer patients, higher 25(OH)D levels correlate with lower cancer incidence and mortality rates. Specifically, each 20 nmol/L increase in serum 25(OH)D is associated with a 12% reduction in colorectal disease-specific mortality and a 7% reduction in all-cause mortality (125). Therefore, accumulating evidence indicates that maintaining serum 25(OH)D levels within the range of 75–100 nmol/L maximizes the risk reduction for colorectal cancer (126). Based on this, a stepwise dosing regimen guided by baseline 25(OH)D levels has been clinically established: for patients with vitamin D deficiency (≤20 ng/mL), an initial dose of 50,000 IU/week is administered for 8 weeks, followed by a maintenance dose of 1,000–2,000 IU/day; for those with insufficiency (21–29 ng/mL), a daily dose of 1,000–2,000 IU is given directly; for those with sufficiency (≥30 ng/mL), routine supplementation is unnecessary. This strategy enables approximately 90% of patients to reach the target level (≥30 ng/mL) within 6 months, with an adverse reaction rate of less than 1%, fully demonstrating the clinical value of biomarker-guided precision dosing in improving efficacy and ensuring safety (119, 127).

**Table 1:** Clinical research summarization of thyroid cancer and vitamin D levels.

Research subject	Vitamin D levels		Conclusions and findings	Article source
	Predetermined thresholds	Experimental results		
70 patients with thyroid cancer	25(OH)D< 30 ng/mL	The median and the range are 17.5(5.9-22.7ng/mL)	Compared with the patients in the healthy control group and the group of patients with benign thyroid nodules, the serum 25(OH)D level of patients with thyroid cancer was significantly lower.	(106)
60 patients with benign thyroid nodules	25(OH)D< 30 ng/mL	The median and the range are 18.7(16.0-23.8 ng/mL)		
60 cases in the healthy control group	25(OH)D< 30 ng/mL	The median and the range are 19.9(16.1-22.4 ng/mL)		
147 patients with differentiated thyroid cancer (114 cases of papillary thyroid carcinoma (PTC) and 33 cases of follicular thyroid carcinoma (FTC))	25(OH)D<20ng/mL	25(OH)D deficiency was observed in 69 PTC patients and 22 FTC patients.	Among patients with PTC and FTC, the proportions of those with 25(OH)D deficiency or insufficiency (< 20 ng/mL) were 66.7% and 60.5% respectively, while in the control group, it was 57.9%. In contrast, there was no significant difference in the 25(OH)D concentration between the patients and the healthy controls.	(109)
57 healthy control subjects(HC)	25(OH)D<20ng/mL	25(OH)D deficiency was found in 33 cases.		
227 patients with thyroid cancer	25(OH)D< 30 ng/mL	25(OH)D deficiency was found in 192 cases.	The proportions of 25(OH)D deficiency in patients with thyroid cancer and the control group were 84.58% and 88.46% respectively. There was no significant difference in the 25(OH)D levels between the patients with thyroid cancer and the control subjects.	(108)
104 cases in the healthy control group	25(OH)D< 30 ng/mL	25(OH)D deficiency was found in 92 cases.		
12 patients with vitamin D deficiency (VDD)	25 (OH) D<37.5 nmol/L	9 cases were thyroid malignancies	The malignancy rates in the VDD group and the VDS group were 75% and 37.5%, respectively. With a p - value of 0.03, it indicates that thyroid cancer is more common in patients with 25(OH)D deficiency.	(107)
88 patients with sufficient vitamin D (VDS)	25 (OH) D>37.5 nmol/L	33 cases were thyroid malignancies		
235 patients with thyroid cancer	25 (OH) D<20 ng/mL	25(OH)D deficiency was found in 166 cases.	The level of 25(OH)D was significantly decreased in 70.6% of patients with thyroid cancer, while 59.3% of the patients in the control group had 25(OH)D deficiency. The statistical result of p = 0.026 indicates that the data is meaningful.	(104)
108 cases in the healthy control group	25 (OH) D<20 ng/mL	25(OH)D deficiency was found in 64 cases.		
53 patients with thyroid cancer	25(OH)D< 30 ng/mL	25(OH)D deficiency was found in 33 cases.	The proportions of 25(OH)D deficiency in patients with thyroid cancer and the control group were 62.26% and 68.81% respectively. There was no significant difference in the 25(OH)D levels between the patients with thyroid cancer and the control subjects.	(110)
5133 cases in the healthy control group	25(OH)D< 30 ng/mL	25(OH)D deficiency was found in 3532 cases.		
85 patients with thyroid cancer	25(OH)D< 20 ng/ml	25(OH)D deficiency was found in 30 cases.	The proportions of 25(OH)D deficiency in patients with thyroid cancer and the control group were 35.3% and 29.4% respectively. There was no significant difference in the 25(OH)D levels between the patients with thyroid cancer and the control subjects.	(111)
85 cases in the healthy control group	25(OH)D< 20 ng/ml	25(OH)D deficiency was found in 25 cases.		

In addition, vitamin D supplementation has been found to have a potential positive effect on thyroid function. Research in this area has been focused primarily in patients with autoimmune thyroid disease, with multiple clinical studies reporting low vitamin D levels in individuals affected by these disorders. These findings suggest a possible link between vitamin D insufficiency and thyroid autoimmunity (**Table 2**). Earlier studies have demonstrated a significantly higher incidence of AT (Autoimmune Thyroid disease) in patients with vitamin D deficiency, likely due to the association between reduced vitamin D levels and elevated thyroid-stimulating antibodies (TSAb), as well as goiter formation (128–132) (**Table 2**). Numerous researches have reported a significant inverse correlation between antithyroid peroxidase antibodies (TPOAb), antithyroglobulin antibodies (TgAb), and serum 25-hydroxyvitamin D (133) (**Table 3**) (134, 135). Notably, individuals with vitamin D deficiency exhibit a higher prevalence of positive TPOAb titers. Moreover, a study has found that after 12 months of vitamin D supplement, participants who received the supplement had significantly lower levels of TPOAb, TgAb, thyrotropin, thyroid hormone, and thyroglobulin in their serum (136), and a notable decrease in the dimensions of both thyroid lobes (137) (**Table 4**) (136, 138–140). Therefore, short-term high-dose oral vitamin D supplementation has been shown to reduce TPOAb and TgAb titers, suggesting its potential as a novel approach to improving thyroid function. However, there are currently no universal guidelines for vitamin D supplementation in patients with thyroid disorders. Moreover, significant variations exist in the dosage and treatment duration across different research cohorts, resulting in a lack of unified reference standards for clinical practice (141). For instance, study designs vary significantly in vitamin D dosage and intervention duration, impeding cross-study comparisons. Moreover, marked interindividual differences exist among participants of different ages and regional origins, contributing to the heterogeneity of experimental results (142). This heterogeneity is not only attributable to the diversity of study designs, but also linked to factors such as thyroid disease subtypes, comorbidities, and physical constitution, which significantly impairs the reliability of therapeutic evidence and its value for clinical translation. Furthermore, VDR expression in individuals is modulated by genetic and environmental factors. Thus, it is hypothesized that genetic variations in the VDR gene may lead to differential responses to vitamin D supplementation. A study investigating the effects of VDR gene polymorphisms indicated that the variant allele of VDR TaqI polymorphism and the FF genotype of VDR FokI polymorphism are associated with better responses to vitamin D supplementation, whereas the VDR BsmI and ApaI polymorphisms show no correlation with such responses (143). However, this study has certain limitations, mainly true genetic heterogeneity between different samples and incomplete data in genetic association research. Clinically, long-term excessive vitamin D supplementation may trigger a series of adverse reactions. Moreover, owing to metabolic disorders, patients with thyroid diseases are more susceptible to such toxicity, and excessive vitamin D intake elevates the risk of hypercalcemic toxicity. Therefore, the safety of vitamin D supplementation therapy deserves particular attention, and future researchers need to conduct large-sample, multicenter prospective cohort studies to define the safe dosage range of vitamin D supplementation for patients with different types of thyroid diseases.

**Table 2:** Clinical research summarization on the relationship between autoimmune thyroid diseases and vitamin D levels.

Research subject	Vitamin D levels		Conclusions and findings	Article source
	Predetermined thresholds	Experimental results		
161 patient with HT	25(OH) D< 30 ng/mL	25(OH)D deficiency was found in 148 cases.	The proportions of 25(OH)D deficiency in patients with HT and the control group were 92% and 63% respectively. The P-value in statistics was less than 0.0001, indicating that the proportion of patients with vitamin D deficiency was higher among those with HT.	(128)
162 cases in the healthy control group	25(OH) D< 30 ng/mL	25(OH)D deficiency was found in 102 cases.		
25(OH)D deficiency was found in 116 cases.	25(OH) D< 20 ng/mL	33 patient had autoimmune thyroid disease AITD.	The prevalence rates of AITD among patients with vitamin D deficiency and those with sufficient vitamin D levels were 28% and 8% respectively. With a statistical p-value of 0.002, it was found that the incidence of AT was significantly higher in patients with vitamin D deficiency.	(129)
25(OH)D deficiency was found in 52 cases.	25(OH) D≥ 20 ng/mL	5 patient had autoimmune thyroid disease AITD.		
26 patients with GD	25(OH)D< 15 ng/ml	25(OH)D deficiency was found in 17 cases.	Compared with the control group, the serum 25(OH)D level in patients with GD was significantly lower. The prevalence of vitamin D deficiency in GD patients was 65.4%, while that in the control group was 32.4%. With a statistical P-value less than 0.05, it was found that the prevalence of vitamin D deficiency in GD patients was significantly higher.	(130)
46 cases in the healthy control group	25(OH)D< 15 ng/ml	25(OH)D deficiency was found in 15 cases.		
70 patients with GD	25(OH)D<50nmol/L	25(OH)D deficiency was found in 65 cases.	The proportion of vitamin D deficiency was 92.86% in patients with GD, 94.29% in patients with HT, and 77.14% in the control group. With a statistical P-value of 0.002, it indicates that compared with the control group, the prevalence rates of vitamin D deficiency in patients with GD and HT are significantly higher.	(131)
70 patient with HT	25(OH)D<50nmol/L	25(OH)D deficiency was found in 66 cases.		
70 cases in the healthy control group	25(OH)D<50nmol/L	25(OH)D deficiency was found in 54 cases.		
50 patient with AITD	25(OH)D< 25 nmol/L	25(OH)D deficiency was found in 36 cases.	The proportion of vitamin D deficiency is 72% among patients with AITD and 30.6% in the control group. With a statistical P-value of less than 0.001, it indicates that compared with the control group, the prevalence of vitamin D deficiency in AITD patients is significantly higher.	(132)
98 cases in the healthy control group	25(OH)D< 25 nmol/L	25(OH)D deficiency was found in 30 cases.		

**Table 3:** Clinical research summarization of the correlation between vitamin D levels and TPOAb and TgAb.

Vitamin D levels	Positive for TPOAb	Positive for TgAb	Conclusions and findings	Article source
25(OH)D<50nmol/L	148 subjects	166 subjects	Compared with those with sufficient 25(OH)D levels, individuals with 25(OH)D deficiency had an increased positive rate of TPOAb and TgAb, and the statistical P-values were both less than 0.05. This indicates that the serum 25(OH)D concentration is negatively correlated with the titer of thyroid autoantibodies.	(134)
25(OH)D>50nmol/L	89 subjects	88 subjects		
25(OH)D< 20 ng/ml	The median is 170.9IU/ml	The median is 40.4IU/ml	Compared with AITD patients with sufficient vitamin D levels, patients in the vitamin D deficiency group had significantly higher positive levels of TgAb and TPOAb.	(135)

**Table 4:** Summary of clinical studies on the supplementation of vitamin D and the levels of TPOAb and TgAb.

Research subject	Vitamin D supplementation program	Target vitamin D level	TPOAb	TgAb	Conclusions and findings	Article source
11,017 subjects	Supplement with 4000 IU per day for 12 months	25(OH)D≥100 nmol/L	down	down	After 12 months of vitamin D supplementation, the levels of TPOAb and TgAb in the participants significantly decreased.	(136)
218 patient with HT	At a dosage of 1200 – 4000 IU per day for 4 consecutive months	25(OH)D≥40ng/mL	down	down	After four months of vitamin D supplementation, the levels of TPOAb and TgAb in the participants significantly decreased.	(138)
102 patient with AITD	At a dosage of 60,000 IU per week and 500 mg per day, for a duration of 8 weeks	25(OH)D≥75 nmol/L	down	unmeasured	After taking vitamin D supplements, the level of TPOAb in the participants significantly decreased.	(139)
46 patient with AITD	At a dosage of 1000 IU per day for 1 month	25(OH)D≥20ng/mL	down	down	After the administration of vitamin D supplements, the levels of TPOAb and TgAb in the participants significantly decreased.	(140)

## 6 Conclusion and future perspectives

VDRs are widely distributed in most human tissues and cells. Beyond its role in bone metabolism and calcium-phosphorus homeostasis, vitamin D plays a significant role in autoimmune diseases, cancer, and metabolic syndromes. In this review, we have explored the association between vitamin D and thyroid disorders, together with its underlying mechanisms of action. Additionally, we analyzed the possible protective effects of vitamin D supplementation on thyroid cancer and evaluated its impact on thyroid function. Based on the analysis of the aforementioned studies, vitamin D exerts a beneficial effect on thyroid cancer progression by directly inhibiting the proliferation, invasion, and metastasis of malignant thyroid cells through interactions involving VDR-mediated signaling pathways, genetic regulation, epigenetic modulation, and transcription factor regulation. Meanwhile, vitamin D can also modulate the immune system and suppress the immune response involved in thyroid autoimmune diseases, thus reducing the disease incidence and improving the function of the thyroid gland. Furthermore, vitamin D can indirectly inhibit the incidence of thyroid autoimmune diseases and thyroid cancer by regulating the homeostasis of intestinal flora through the thyroid-endocrine-intestinal axis. (**Table 5**) Consequently, vitamin D deficiency is increasingly recognized as a risk factor for a range of thyroid diseases, including autoimmune thyroid disorders and thyroid cancer.

**Table 5:** Summary table of molecular pathways, biomarkers, and vitamin D correlations in thyroid-related diseases.

Disease category	Major related molecular targets/Pathways	Biomarkers	Correlation with Vitamin D
Thyroid Cancer (TC)	—Receptors: VDR/RXR heterodimer —Signaling pathways: Wnt/β-catenin, Ras-MEK-ERK, PTEN/PI3K-AKT, NF-κB —Cell cycle regulation: C-MYC, p27 —Epigenetics: Histone modification, DNA methyltransferases (DNMTs), miRNA expression (miR-34a, let-7, etc.) —Metastasis-related: EMT regulators (E-cadherin, Snail, Slug),CXCL8/CCL2 chemokines —Metabolic enzymes: CYP27B1, CYP24A1	—Serum biomarkers: Decreased 25(OH)D level —Genetic biomarkers: VDR gene polymorphisms (FokI, TaqI, BsmI) — Cancer stem cell markers (Oct4, Nanog, Sox2)	Vitamin D inhibits tumor cell proliferation, induces apoptosis, blocks metastasis through VDR-mediated signaling pathways, and influences the development of thyroid cancer by regulating epigenetic gene expression. Additionally, low vitamin D levels are associated with an increased risk of thyroid cancer.
Autoimmune Thyroid Diseases (AITDs)	—Receptors: VDR/RXR heterodimer —Immune regulatory pathways: Th1/Th17/Treg/Th2 cell balance, NF-κB inflammatory pathway —Target cells: Dendritic cells (DCs), B cells, T cells	—Serum biomarkers: Decreased 25(OH)D level —Immune biomarkers: Increased TPOAb, TgAb, TRAb —Imbalanced Th17/Treg ratio —Increased inflammatory cytokines (TNF-α, IL-6, IL-17, IFN-γ)	Vitamin D regulates the differentiation and function of immune cells, inhibits autoantibodies, and alleviates thyroid autoimmune responses. Furthermore, vitamin D deficiency is associated with an increased incidence of AITDs.
Intestinal Microbiota-Related Thyroid Dysfunction	— Intestinal barrier regulation: Tight junction (TJ) proteins —Metabolic pathways: Short-chain fatty acid (SCFA) synthesis pathway —Axis regulation: Thyroid-endocrine-intestinal axis	—Microbiota biomarkers: Decreased abundance of beneficial bacteria, reduced microbiota diversity —Serum biomarkers: 25(OH)D, T3/T4, TSH —Increased intestinal permeability	Vitamin D can regulate intestinal microbiota homeostasis, protect intestinal barrier integrity, and indirectly improve thyroid function through the thyroid-endocrine-intestinal axis. Moreover, vitamin D deficiency may lead to intestinal microbiota dysbiosis, which in turn induces thyroid diseases.

The pleiotropic effects of vitamin D have been demonstrated in numerous studies, providing evidence of its protective role against thyroid carcinoma and the inhibitory effects of pharmacological 1,25(OH)_2_D or its analogs on thyroid cancer cell proliferation. Meanwhile, nearly all studies investigating the impact of vitamin D supplementation on thyroid function have reported reduced levels of anti-thyroid antibodies following vitamin D supplementation. However, the limited number of published intervention studies in this field has resulted in ongoing controversy surrounding the preventive and therapeutic potential of vitamin D or its analogs in thyroid diseases. To substantiate this view, numerous large-scale multicenter studies are needed to evaluate the clinical impact of vitamin D supplementation on thyroid disease. Furthermore, this article also elaborates on interindividual differences in the personalized treatment of nutritional vitamin D and highlights strategies for translating precision medicine into clinical practice. However, the optimal dosage of vitamin D supplements remains to be determined, and further investigations via observational studies and long-term follow-up randomized controlled trials are required to fully validate its potential for precision therapy in thyroid diseases.

## References

[1] Pitoia F Trimboli P . New insights in thyroid diagnosis and treatment. Rev Endocr Metab Disord. (2024) 25:1–3. doi: 10.1007/s11154-023-09859-5, PMID: 38041785 PMC10808208

[2] Vargas-Uricoechea H . Molecular mechanisms in autoimmune thyroid disease. Cells. (2023) 12(6):918. doi: 10.3390/cells12060918, PMID: 36980259 PMC10047067

[3] Saini S Rathore A Suresh N Singh V . B, C, D, E, and K as molecular modulators: Integrative roles in precision medicine, nutrigenomics, therapy, and disease prevention. J Precis Med Mol Med. (2025) 1:44–52. doi: 10.4103/IJPMMM.IJPMMM_10_25

[4] Muscogiuri G Tirabassi G Bizzaro G Orio F Paschou SA Vryonidou A . Vitamin D and thyroid disease: to D or not to D? Eur J Clin Nutr. (2015) 69:291–6. doi: 10.1038/ejcn.2014.265, PMID: 25514898

[5] Bikle D . Nonclassic actions of vitamin D. J Clin Endocrinol Metab. (2009) 94:26–34. doi: 10.1210/jc.2008-1454, PMID: 18854395 PMC2630868

[6] D'Aurizio F Villalta D Metus P Doretto P Tozzoli R . Is vitamin D a player or not in the pathophysiology of autoimmune thyroid diseases? Autoimmun Rev. (2015) 14:363–9. doi: 10.1016/j.autrev.2014.10.008, PMID: 25308530

[7] Feldman D Krishnan AV Swami S Giovannucci E Feldman BJ . The role of vitamin D in reducing cancer risk and progression. Nat Rev Cancer. (2014) 14:342–57. doi: 10.1038/nrc3691, PMID: 24705652

[8] Clinckspoor I Verlinden L Mathieu C Bouillon R Verstuyf A Decallonne B . Vitamin D in thyroid tumorigenesis and development. Prog Histochem Cytochem. (2013) 48:65–98. doi: 10.1016/j.proghi.2013.07.001, PMID: 23890557

[9] Hu Y Xue C Ren S Dong L Gao J Li X . Association between vitamin D status and thyroid cancer: a meta-analysis. Front Nutr. (2024) 11:1423305. doi: 10.3389/fnut.2024.1423305, PMID: 38962442 PMC11221265

[10] Fitzgerald SP Bean NG Falhammar H Hoermann R . Physiological linkage of thyroid and pituitary sensitivities. Endocrine. (2023) 79:143–51. doi: 10.1007/s12020-022-03184-8, PMID: 36115005 PMC9813051

[11] Dias Lopes NM Mendonca Lens HH Armani A Marinello PC Cecchini AL . Thyroid cancer and thyroid autoimmune disease: A review of molecular aspects and clinical outcomes. Pathol Res Pract. (2020) 216:153098. doi: 10.1016/j.prp.2020.153098, PMID: 32825964

[12] Antonelli A Ferrari SM Ragusa F Elia G Paparo SR Ruffilli I . Graves' disease: Epidemiology, genetic and environmental risk factors and viruses. Best Pract Res Clin Endocrinol Metab. (2020) 34:101387. doi: 10.1016/j.beem.2020.101387, PMID: 32107168

[13] Liu J Xu T Ma L Chang W . Signal pathway of estrogen and estrogen receptor in the development of thyroid cancer. Front Oncol. (2021) 11:593479. doi: 10.3389/fonc.2021.593479, PMID: 33996538 PMC8113849

[14] Boucai L Zafereo M Cabanillas ME . Thyroid cancer: A review. Jama. (2024) 331:425–35. doi: 10.1001/jama.2023.26348, PMID: 38319329

[15] Chen DW Lang BHH McLeod DSA Newbold K Haymart MR . Thyroid cancer. Lancet. (2023) 401:1531–44. doi: 10.1016/S0140-6736(23)00020-X, PMID: 37023783

[16] Younis E . Oncogenesis of thyroid cancer. Asian Pac J Cancer Prev. (2017) 18:1191–9. doi: 10.22034/APJCP.2017.18.5.1191, PMID: 28610401 PMC5555522

[17] Prete A Borges de Souza P Censi S Muzza M Nucci N Sponziello M . Update on fundamental mechanisms of thyroid cancer. Front Endocrinol (Lausanne). (2020) 11:102. doi: 10.3389/fendo.2020.00102, PMID: 32231639 PMC7082927

[18] Zhao J Wang H Zhang Z Zhou X Yao J Zhang R . Vitamin D deficiency as a risk factor for thyroid cancer: A meta-analysis of case-control studies. Nutrition. (2019) 57:5–11. doi: 10.1016/j.nut.2018.04.015, PMID: 30086436

[19] Bogusławska J Godlewska M Gajda E Piekiełko-Witkowska A . Cellular and molecular basis of thyroid autoimmunity. Eur Thyroid J. (2022) 11. doi: 10.1530/ETJ-21-0024, PMID: 34981746 PMC9142813

[20] Antonelli A Ferrari SM Corrado A Di Domenicantonio A Fallahi P . Autoimmune thyroid disorders. Autoimmun Rev. (2015) 14:174–80. doi: 10.1016/j.autrev.2014.10.016, PMID: 25461470

[21] Ralli M Angeletti D Fiore M D'Aguanno V Lambiase A Artico M . Hashimoto's thyroiditis: An update on pathogenic mechanisms, diagnostic protocols, therapeutic strategies, and potential Malignant transformation. Autoimmun Rev. (2020) 19:102649. doi: 10.1016/j.autrev.2020.102649, PMID: 32805423

[22] Ragusa F Fallahi P Elia G Gonnella D Paparo SR Giusti C . Hashimotos' thyroiditis: Epidemiology, pathogenesis, clinic and therapy. Best Pract Res Clin Endocrinol Metab. (2019) 33:101367. doi: 10.1016/j.beem.2019.101367, PMID: 31812326

[23] Lebiedziński F Lisowska KA . Impact of vitamin D on immunopathology of hashimoto's thyroiditis: from theory to practice. Nutrients. (2023) 15. doi: 10.3390/nu15143174, PMID: 37513592 PMC10385100

[24] Kahaly GJ . Management of graves thyroidal and extrathyroidal disease: an update. J Clin Endocrinol Metab. (2020) 105:3704–20. doi: 10.1210/clinem/dgaa646, PMID: 32929476 PMC7543578

[25] Petranović Ovčariček P Görges R Giovanella L . Autoimmune thyroid diseases. Semin Nucl Med. (2024) 54:219–36. doi: 10.1053/j.semnuclmed.2023.11.002, PMID: 38044176

[26] Kim D . The role of vitamin D in thyroid diseases. Int J Mol Sci. (2017) 18(9):1949. doi: 10.3390/ijms18091949, PMID: 28895880 PMC5618598

[27] Płudowski P Kos-Kudła B Walczak M Fal A Zozulińska-Ziółkiewicz D Sieroszewski P . Guidelines for preventing and treating vitamin D deficiency: A 2023 update in Poland. Nutrients. (2023) 15. doi: 10.3390/nu15030695, PMID: 36771403 PMC9920487

[28] Makariou S Liberopoulos EN Elisaf M Challa A . Novel roles of vitamin D in disease: what is new in 2011? Eur J Intern Med. (2011) 22:355–62., PMID: 21767752 10.1016/j.ejim.2011.04.012

[29] Plum LA DeLuca HF . Vitamin D, disease and therapeutic opportunities. Nat Rev Drug Discov. (2010) 9:941–55. doi: 10.1038/nrd3318, PMID: 21119732

[30] Delrue C Speeckaert MM . Vitamin D and vitamin D-binding protein in health and disease. Int J Mol Sci. (2023) 24(5):4642. doi: 10.3390/ijms24054642, PMID: 36902073 PMC10003016

[31] Demay MB Pittas AG Bikle DD Diab DL Kiely ME Lazaretti-Castro M . Vitamin D for the prevention of disease: an endocrine society clinical practice guideline. J Clin Endocrinol Metab. (2024) 109:1907–47. doi: 10.1210/clinem/dgae290, PMID: 38828931

[32] Carlberg C Velleuer E . Vitamin D and the risk for cancer: A molecular analysis. Biochem Pharmacol. (2022) 196:114735. doi: 10.1016/j.bcp.2021.114735, PMID: 34411566

[33] Carlberg C Raczyk M Zawrotna N . Vitamin D: A master example of nutrigenomics. Redox Biol. (2023) 62:102695. doi: 10.1016/j.redox.2023.102695, PMID: 37043983 PMC10119805

[34] Salehi-Tabar R Nguyen-Yamamoto L Tavera-Mendoza LE Quail T Dimitrov V An BS . Vitamin D receptor as a master regulator of the c-MYC/MXD1 network. Proc Natl Acad Sci U.S.A. (2012) 109:18827–32. doi: 10.1073/pnas.1210037109, PMID: 23112173 PMC3503153

[35] Chiang KC Kuo SF Chen CH Ng S Lin SF Yeh CN . MART-10, the vitamin D analog, is a potent drug to inhibit anaplastic thyroid cancer cell metastatic potential. Cancer Lett. (2015) 369:76–85. doi: 10.1016/j.canlet.2015.07.024, PMID: 26282787

[36] Zhang W Ruan X Huang Y Zhang W Xu G Zhao J . SETMAR facilitates the differentiation of thyroid cancer by regulating SMARCA2-mediated chromatin remodeling. Advanced Sci (Weinheim Baden-Wurttemberg Germany). (2024) 11:e2401712. doi: 10.1002/advs.202401712, PMID: 38900084 PMC11348079

[37] So JY Suh N . Targeting cancer stem cells in solid tumors by vitamin D. J Steroid Biochem Mol Biol. (2015) 148:79–85. doi: 10.1016/j.jsbmb.2014.10.007, PMID: 25460302 PMC4361233

[38] Hu PS Li T Lin JF Qiu MZ Wang DS Liu ZX . VDR-SOX2 signaling promotes colorectal cancer stemness and Malignancy in an acidic microenvironment. Signal transduction targeted Ther. (2020) 5:183. doi: 10.1038/s41392-020-00230-7, PMID: 32900990 PMC7479104

[39] Takebe N Miele L Harris PJ Jeong W Bando H Kahn M . Targeting Notch, Hedgehog, and Wnt pathways in cancer stem cells: clinical update. Nat Rev Clin Oncol. (2015) 12:445–64. doi: 10.1038/nrclinonc.2015.61, PMID: 25850553 PMC4520755

[40] Ling Y Xu F Xia X Dai D Sun R Xie Z . Vitamin D receptor regulates proliferation and differentiation of thyroid carcinoma via the E-cadherin-β-catenin complex. J Mol Endocrinol. (2022) 68:137–51. doi: 10.1530/JME-21-0167, PMID: 35099410 PMC8942331

[41] Ricca C Aillon A Viano M Bergandi L Aldieri E Silvagno F . Vitamin D inhibits the epithelial-mesenchymal transition by a negative feedback regulation of TGF-β activity. J Steroid Biochem Mol Biol. (2019) 187:97–105. doi: 10.1016/j.jsbmb.2018.11.006, PMID: 30465855

[42] Xiong XR Tian XL Huo RJ Dong YT Liu D Bai JC . 1α, 25-dihydroxyvitamin D3 inhibits transforming growth factor β1-induced epithelial-mesenchymal transition via β-catenin pathway. Chin Med J. (2020) 133:1298–303. doi: 10.1097/CM9.0000000000000830, PMID: 32452895 PMC7289296

[43] Liu W Asa SL Fantus IG Walfish PG Ezzat S . Vitamin D arrests thyroid carcinoma cell growth and induces p27 dephosphorylation and accumulation through PTEN/akt-dependent and -independent pathways. Am J Pathol. (2002) 160:511–9. doi: 10.1016/S0002-9440(10)64870-5, PMID: 11839571 PMC1850654

[44] Liu W Asa SL Ezzat S . 1alpha,25-Dihydroxyvitamin D3 targets PTEN-dependent fibronectin expression to restore thyroid cancer cell adhesiveness. Mol Endocrinol. (2005) 19:2349–57. doi: 10.1210/me.2005-0117, PMID: 15890670

[45] Bessler H Djaldetti M . 1α,25-Dihydroxyvitamin D3 modulates the interaction between immune and colon cancer cells. BioMed Pharmacother. (2012) 66:428–32. doi: 10.1016/j.biopha.2012.06.005, PMID: 22795808

[46] Dıaz L Dıaz-Muñoz M Garcıa-Gaytan AC Méndez I . Mechanistic effects of calcitriol in cancer biology. Nutrients. (2015) 7:5020–50. doi: 10.3390/nu7065020, PMID: 26102214 PMC4488829

[47] Pang R Xu Y Hu X Liu B Yu J . Vitamin D receptor knockdown attenuates the antiproliferative, pro−apoptotic and anti−invasive effect of vitamin D by activating the Wnt/β−catenin signaling pathway in papillary thyroid cancer. Mol Med Rep. (2020) 22:4135–42. doi: 10.3892/mmr.2020.11522, PMID: 33000217 PMC7533458

[48] Zhang T Zhang H He L Wang Z Dong W Sun W . Potential use of 1-25-dihydroxyvitamin D in the diagnosis and treatment of papillary thyroid cancer. Med Sci Monit. (2018) 24:1614–23. doi: 10.12659/MSM.909544, PMID: 29553126 PMC5872905

[49] Kim MJ Kim D Koo JS Lee JH Nam KH . Vitamin D receptor expression and its clinical significance in papillary thyroid cancer. Technol Cancer Res Treat. (2022) 21:15330338221089933. doi: 10.1177/15330338221089933, PMID: 35379049 PMC8988685

[50] Li B Lv L Li W . 1,25-Dihydroxy vitamin D3 inhibits the Ras-MEK-ERK pathway and regulates proliferation and apoptosis of papillary thyroid carcinoma. Steroids. (2020) 159:108585. doi: 10.1016/j.steroids.2020.108585, PMID: 31982425

[51] Deeb KK Trump DL Johnson CS . Vitamin D signalling pathways in cancer: potential for anticancer therapeutics. Nat Rev Cancer. (2007) 7:684–700. doi: 10.1038/nrc2196, PMID: 17721433

[52] Evans RM . The steroid and thyroid hormone receptor superfamily. Sci (New York NY). (1988) 240:889–95. doi: 10.1126/science.3283939, PMID: 3283939 PMC6159881

[53] Snegarova V Naydenova D . Vitamin D: a review of its effects on epigenetics and gene regulation. Folia Med. (2020) 62:662–7. doi: 10.3897/folmed.62.e50204., PMID: 33415918

[54] Carlberg C . Nutrigenomics of vitamin D. Nutrients. (2019) 11(3):676. doi: 10.3390/nu11030676, PMID: 30901909 PMC6470874

[55] Kamen DL Tangpricha V . Vitamin D and molecular actions on the immune system: modulation of innate and autoimmunity. J Mol Med (Berlin Germany). (2010) 88:441–50. doi: 10.1007/s00109-010-0590-9, PMID: 20119827 PMC2861286

[56] Maciejewski A Lacka K . Vitamin D-related genes and thyroid cancer-A systematic review. Int J Mol Sci. (2022) 23(21):13661. doi: 10.3390/ijms232113661, PMID: 36362448 PMC9658610

[57] Kowalowka M Gorna I Karazniewicz-Łada M Kusyk D Przysławski J Drzymała-Czyż S . Nutri-epigenetic regulation of vitamin D-impact on metabolism and biological functions: narrative review. Metabolites. (2025) 15(7):436. doi: 10.3390/metabo15070436, PMID: 40710536 PMC12299634

[58] Beysel S Eyerci N Pinarli FA Apaydin M Kizilgul M Caliskan M . VDR gene FokI polymorphism as a poor prognostic factor for papillary thyroid cancer. Tumour biology: J Int Soc Oncodevelopmental Biol Med. (2018) 40:1010428318811766. doi: 10.1177/1010428318811766, PMID: 30486759

[59] Cocolos AM Muresan A Caragheorgheopol A Ghemigian M Ioachim D Poiana C . Vitamin D status and VDR polymorphisms as prognostic factors in differentiated thyroid carcinoma. In Vivo (Athens Greece). (2022) 36:2434–41. doi: 10.21873/invivo.12977, PMID: 36099120 PMC9463930

[60] Coperchini F Greco A Croce L Petrosino E Grillini B Magri F . Vitamin D reduces thyroid cancer cells migration independently from the modulation of CCL2 and CXCL8 chemokines secretion. Front Endocrinol (Lausanne). (2022) 13:876397. doi: 10.3389/fendo.2022.876397, PMID: 35498406 PMC9044905

[61] Wang TJ Pencina MJ Booth SL Jacques PF Ingelsson E Lanier K . Vitamin D deficiency and risk of cardiovascular disease. Circulation. (2008) 117:503–11. doi: 10.1161/CIRCULATIONAHA.107.706127, PMID: 18180395 PMC2726624

[62] Wimalawansa SJ . Infections and autoimmunity-the immune system and vitamin D: A systematic review. Nutrients. (2023) 15(17):3842. doi: 10.3390/nu15173842, PMID: 37686873 PMC10490553

[63] Aranow C . Vitamin D and the immune system. J Investig Med. (2011) 59:881–6. doi: 10.2310/JIM.0b013e31821b8755, PMID: 21527855 PMC3166406

[64] Bhalla AK Amento EP Serog B Glimcher LH . 1,25-Dihydroxyvitamin D3 inhibits antigen-induced T cell activation. J Immunol. (1984) 133:1748–54. doi: 10.4049/jimmunol.133.4.1748, PMID: 6206136

[65] Mattner F Smiroldo S Galbiati F Muller M Di Lucia P Poliani PL . Inhibition of Th1 development and treatment of chronic-relapsing experimental allergic encephalomyelitis by a non-hypercalcemic analogue of 1,25-dihydroxyvitamin D(3). Eur J Immunol. (2000) 30:498–508. doi: 10.1002/1521-4141(200002)30:2<498::AID-IMMU498>3.0.CO;2-Q, PMID: 10671205

[66] Boonstra A Barrat FJ Crain C Heath VL Savelkoul HF O'Garra A . 1alpha,25-Dihydroxyvitamin d3 has a direct effect on naive CD4(+) T cells to enhance the development of Th2 cells. J Immunol. (2001) 167:4974–80. doi: 10.4049/jimmunol.167.9.4974, PMID: 11673504

[67] Prietl B Treiber G Pieber TR Amrein K . Vitamin D and immune function. Nutrients. (2013) 5:2502–21. doi: 10.3390/nu5072502, PMID: 23857223 PMC3738984

[68] Mazur A Frączek P Tabarkiewicz J . Vitamin D as a nutri-epigenetic factor in autoimmunity-A review of current research and reports on vitamin D deficiency in autoimmune diseases. Nutrients. (2022) 14(20):4286. doi: 10.3390/nu14204286, PMID: 36296970 PMC9611618

[69] Bscheider M Butcher EC . Vitamin D immunoregulation through dendritic cells. Immunology. (2016) 148:227–36. doi: 10.1111/imm.12610, PMID: 27040466 PMC4913286

[70] Cantorna MT Snyder L Lin YD Yang L . Vitamin D and 1,25(OH)2D regulation of T cells. Nutrients. (2015) 7:3011–21. doi: 10.3390/nu7043011, PMID: 25912039 PMC4425186

[71] Durá-Travé T Gallinas-Victoriano F . Autoimmune thyroiditis and vitamin D. Int J Mol Sci. (2024) 25(6):3154. doi: 10.3390/ijms25063154, PMID: 38542128 PMC10969999

[72] Cyprian F Lefkou E Varoudi K Girardi G . Immunomodulatory effects of vitamin D in pregnancy and beyond. Front Immunol. (2019) 10:2739. doi: 10.3389/fimmu.2019.02739, PMID: 31824513 PMC6883724

[73] Mele C Caputo M Bisceglia A Samà MT Zavattaro M Aimaretti G . Immunomodulatory effects of vitamin D in thyroid diseases. Nutrients. (2020) 12. doi: 10.3390/nu12051444, PMID: 32429416 PMC7284826

[74] Zhao R Zhang W Ma C Zhao Y Xiong R Wang H . Immunomodulatory function of vitamin D and its role in autoimmune thyroid disease. Front Immunol. (2021) 12:574967. doi: 10.3389/fimmu.2021.574967, PMID: 33679732 PMC7933459

[75] Sassi F Tamone C D'Amelio P . Vitamin D: nutrient, hormone, and immunomodulator. Nutrients. (2018) 10(11):1656. doi: 10.3390/nu10111656, PMID: 30400332 PMC6266123

[76] Rolf L Muris AH Hupperts R Damoiseaux J . Vitamin D effects on B cell function in autoimmunity. Ann N Y Acad Sci. (2014) 1317:84–91. doi: 10.1111/nyas.12440, PMID: 24761763

[77] Zhang Q He X Chen W Jiu J Gao C Gao T . Vitamin D3 attenuates autoimmune thyroiditis by regulating Th17/Treg cell differentiation via YAP/JAK1/STAT1 axis. Immunol Lett. (2024) 269:106890. doi: 10.1016/j.imlet.2024.106890, PMID: 38959983

[78] Mosmann TR Cherwinski H Bond MW Giedlin MA Coffman RL . Two types of murine helper T cell clone. I. Definition according to profiles of lymphokine activities and secreted proteins. J Immunol. (1986) 136:2348–57. doi: 10.4049/jimmunol.136.7.2348 2419430

[79] Mosmann TR Coffman RL . TH1 and TH2 cells: different patterns of lymphokine secretion lead to different functional properties. Annu Rev Immunol. (1989) 7:145–73. doi: 10.1146/annurev.iy.07.040189.001045, PMID: 2523712

[80] Antonelli A Fallahi P Elia G Ragusa F Paparo SR Ruffilli I . Graves' disease: Clinical manifestations, immune pathogenesis (cytokines and chemokines) and therapy. Best Pract Res Clin Endocrinol Metab. (2020) 34:101388. doi: 10.1016/j.beem.2020.101388, PMID: 32059832

[81] Baeke F Takiishi T Korf H Gysemans C Mathieu C . Vitamin D: modulator of the immune system. Curr Opin Pharmacol. (2010) 10:482–96. doi: 10.1016/j.coph.2010.04.001, PMID: 20427238

[82] Miraglia Del Giudice M Indolfi C Strisciuglio C . Vitamin D: immunomodulatory aspects. J Clin Gastroenterol. (2018) 52:S86–8. doi: 10.1097/MCG.0000000000001112, PMID: 30300262

[83] Fang H Kang J Zhang D . Microbial production of vitamin B(12): a review and future perspectives. Microb Cell Fact. (2017) 16:15. doi: 10.1186/s12934-017-0631-y, PMID: 28137297 PMC5282855

[84] LeBlanc JG Chain F Martın R Bermúdez-Humarán LG Courau S Langella P . Beneficial effects on host energy metabolism of short-chain fatty acids and vitamins produced by commensal and probiotic bacteria. Microb Cell Fact. (2017) 16:79. doi: 10.1186/s12934-017-0691-z, PMID: 28482838 PMC5423028

[85] Bastiaanssen TFS Cowan CSM Claesson MJ Dinan TG Cryan JF . Making sense of … the microbiome in psychiatry. Int J Neuropsychopharmacol. (2019) 22:37–52. doi: 10.1093/ijnp/pyy067, PMID: 30099552 PMC6313131

[86] Sun M He C Cong Y Liu Z . Regulatory immune cells in regulation of intestinal inflammatory response to microbiota. Mucosal Immunol. (2015) 8:969–78. doi: 10.1038/mi.2015.49, PMID: 26080708 PMC4540654

[87] Gong B Wang C Meng F Wang H Song B Yang Y . Association between gut microbiota and autoimmune thyroid disease: A systematic review and meta-analysis. Front Endocrinol (Lausanne). (2021) 12:774362. doi: 10.3389/fendo.2021.774362, PMID: 34867823 PMC8635774

[88] Jiang W Lu G Gao D Lv Z Li D . The relationships between the gut microbiota and its metabolites with thyroid diseases. Front Endocrinol (Lausanne). (2022) 13:943408. doi: 10.3389/fendo.2022.943408, PMID: 36060978 PMC9433865

[89] Knezevic J Starchl C Tmava Berisha A Amrein K . Thyroid-gut-axis: how does the microbiota influence thyroid function? Nutrients. (2020) 12(6):1769. doi: 10.3390/nu12061769, PMID: 32545596 PMC7353203

[90] Zimmermann MB . The influence of iron status on iodine utilization and thyroid function. Annu Rev Nutr. (2006) 26:367–89. doi: 10.1146/annurev.nutr.26.061505.111236, PMID: 16602928

[91] Beard JL Brigham DE Kelley SK Green MH . Plasma thyroid hormone kinetics are altered in iron-deficient rats. J Nutr. (1998) 128:1401–8. doi: 10.1093/jn/128.8.1401, PMID: 9687562

[92] Fröhlich E Wahl R . Microbiota and thyroid interaction in health and disease. Trends Endocrinol Metab. (2019) 30:479–90. doi: 10.1016/j.tem.2019.05.008, PMID: 31257166

[93] Zhao F Feng J Li J Zhao L Liu Y Chen H . Alterations of the gut microbiota in hashimoto's thyroiditis patients. Thyroid. (2018) 28:175–86. doi: 10.1089/thy.2017.0395, PMID: 29320965

[94] Ishaq HM Mohammad IS Guo H Shahzad M Hou YJ Ma C . Molecular estimation of alteration in intestinal microbial composition in Hashimoto's thyroiditis patients. BioMed Pharmacother. (2017) 95:865–74. doi: 10.1016/j.biopha.2017.08.101, PMID: 28903182

[95] Yu X Jiang W Kosik RO Song Y Luo Q Qiao T . Gut microbiota changes and its potential relations with thyroid carcinoma. J Adv Res. (2022) 35:61–70. doi: 10.1016/j.jare.2021.04.001, PMID: 35003794 PMC8721249

[96] Ooi JH Li Y Rogers CJ Cantorna MT . Vitamin D regulates the gut microbiome and protects mice from dextran sodium sulfate-induced colitis. J Nutr. (2013) 143:1679–86. doi: 10.3945/jn.113.180794, PMID: 23966330 PMC3771816

[97] Tangestani H Boroujeni HK Djafarian K Emamat H Shab-Bidar S . Vitamin D and the gut microbiota: a narrative literature review. Clin Nutr Res. (2021) 10:181–91. doi: 10.7762/cnr.2021.10.3.181, PMID: 34386438 PMC8331286

[98] Liu Q Sun W Zhang H . Interaction of gut microbiota with endocrine homeostasis and thyroid cancer. Cancers (Basel). (2022) 14(11):2656. doi: 10.3390/cancers14112656, PMID: 35681636 PMC9179244

[99] Assa A Vong L Pinnell LJ Avitzur N Johnson-Henry KC Sherman PM . Vitamin D deficiency promotes epithelial barrier dysfunction and intestinal inflammation. J Infect Dis. (2014) 210:1296–305. doi: 10.1093/infdis/jiu235, PMID: 24755435

[100] Kong J Zhang Z Musch MW Ning G Sun J Hart J . Novel role of the vitamin D receptor in maintaining the integrity of the intestinal mucosal barrier. Am J Physiol Gastrointest Liver Physiol. (2008) 294:G208–16. doi: 10.1152/ajpgi.00398.2007, PMID: 17962355

[101] Fasano A Shea-Donohue T . Mechanisms of disease: the role of intestinal barrier function in the pathogenesis of gastrointestinal autoimmune diseases. Nat Clin Pract Gastroenterol Hepatol. (2005) 2:416–22. doi: 10.1038/ncpgasthep0259, PMID: 16265432

[102] Cereijido M Contreras RG Flores-Benıtez D Flores-Maldonado C Larre I Ruiz A . New diseases derived or associated with the tight junction. Arch Med Res. (2007) 38:465–78. doi: 10.1016/j.arcmed.2007.02.003, PMID: 17560451

[103] König J Wells J Cani PD García-Ródenas CL MacDonald T Mercenier A . Human intestinal barrier function in health and disease. Clin Transl Gastroenterol. (2016) 7:e196. doi: 10.1038/ctg.2016.54, PMID: 27763627 PMC5288588

[104] Sahin M Ucan B Ginis Z Topaloğlu O Güngüneş A Bozkurt NÇ . Vitamin D3 levels and insulin resistance in papillary thyroid cancer patients. Med Oncol. (2013) 30:589. doi: 10.1007/s12032-013-0589-5, PMID: 23645546

[105] Kim JR Kim BH Kim SM Oh MY Kim WJ Jeon YK . Low serum 25 hydroxyvitamin D is associated with poor clinicopathologic characteristics in female patients with papillary thyroid cancer. Thyroid: Off J Am Thyroid Assoc. (2014) 24:1618–24. doi: 10.1089/thy.2014.0090, PMID: 25133449

[106] Abdellateif MS Shaarawy S Elesawy YF Mansour M Tharwat E Ibrahim NH . The role of vitamin D, platelet-derived growth factor and insulin-like growth factor 1 in the progression of thyroid diseases. Asian Pac J Cancer Prev. (2020) 21:2083–9. doi: 10.31557/APJCP.2020.21.7.2083, PMID: 32711436 PMC7573424

[107] Roskies M Dolev Y Caglar D Hier MP Mlynarek A Majdan A . Vitamin D deficiency as a potentially modifiable risk factor for thyroid cancer. J Otolaryngol Head Neck Surg. (2012) 41:160–3., PMID: 22762696

[108] Penna-Martinez M Ramos-Lopez E Stern J Kahles H Hinsch N Hansmann ML . Impaired vitamin D activation and association with CYP24A1 haplotypes in differentiated thyroid carcinoma. Thyroid. (2012) 22:709–16. doi: 10.1089/thy.2011.0330, PMID: 22690899 PMC3387756

[109] Penna-Martinez M Ramos-Lopez E Stern J Hinsch N Hansmann ML Selkinski I . Vitamin D receptor polymorphisms in differentiated thyroid carcinoma. Thyroid. (2009) 19:623–8. doi: 10.1089/thy.2008.0388, PMID: 19499989

[110] Choi YM Kim WG Kim TY Bae SJ Kim HK Jang EK . Serum vitamin D3 levels are not associated with thyroid cancer prevalence in euthyroid subjects without autoimmune thyroid disease. Korean J Intern Med. (2017) 32:102–8. doi: 10.3904/kjim.2015.090, PMID: 27581957 PMC5214716

[111] Heidari Z Nikbakht M Mashhadi MA Jahantigh M Mansournia N Sheikhi V . Vitamin D deficiency associated with differentiated thyroid carcinoma: A case- control study. Asian Pac J Cancer Prev. (2017) 18:3419–22. doi: 0.22034/APJCP.2017.18.12.3419, PMID: 29286613 10.22034/APJCP.2017.18.12.3419 PMC5980904

[112] Palanca A Ampudia-Blasco FJ Real JT . The controversial role of vitamin D in thyroid cancer prevention. Nutrients. (2022) 14(13):2593. doi: 10.3390/nu14132593, PMID: 35807774 PMC9268358

[113] Zhang J Zheng S Xie R Zhang J Chen X Xu S . Epigenetic control in thyroid cancer: mechanisms and clinical perspective. Cell Death Discov. (2025) 11:387. doi: 10.1038/s41420-025-02688-2, PMID: 40819127 PMC12357867

[114] Yu X Zhang H Zhang H Hou C Wang X Gu P . The role of epigenetic methylations in thyroid Cancer. World J Surg Oncol. (2024) 22:281. doi: 10.1186/s12957-024-03568-2, PMID: 39456011 PMC11515417

[115] Jurkowska RZ Jurkowski TP Jeltsch A . Structure and function of mammalian DNA methyltransferases. Chembiochem: Eur J Chem Biol. (2011) 12:206–22. doi: 10.1002/cbic.201000195, PMID: 21243710

[116] Ong LTC Booth DR Parnell GP . Vitamin D and its effects on DNA methylation in development, aging, and disease. Mol Nutr Food Res. (2020) 64:e2000437. doi: 10.1002/mnfr.202000437, PMID: 33079481

[117] Keum N Lee DH Greenwood DC Manson JE Giovannucci E . Vitamin D supplementation and total cancer incidence and mortality: a meta-analysis of randomized controlled trials. Ann Oncol. (2019) 30:733–43. doi: 10.1093/annonc/mdz059, PMID: 30796437 PMC6821324

[118] Veeresh PKM Basavaraju CG Dallavalasa S Anantharaju PG Natraj SM Sukocheva OA . Vitamin D3 inhibits the viability of breast cancer cells *in vitro* and ehrlich ascites carcinomas in mice by promoting apoptosis and cell cycle arrest and by impeding tumor angiogenesis. Cancers. (2023) 15(19):4833. doi: 10.3390/cancers15194833, PMID: 37835527 PMC10571758

[119] Dwivedi S Singh V Sen A Yadav D Agrawal R Kishore S . Vitamin D in disease prevention and cure-part I: an update on molecular mechanism and significance on human health. Indian J Clin biochemistry: IJCB. (2025) 40:339–81. doi: 10.1007/s12291-024-01251-7, PMID: 40625600 PMC12229305

[120] Bikle DD . Vitamin D metabolism, mechanism of action, and clinical applications. Chem Biol. (2014) 21:319–29. doi: 10.1016/j.chembiol.2013.12.016, PMID: 24529992 PMC3968073

[121] Zeisel SH . Precision (Personalized) nutrition: understanding metabolic heterogeneity. Annu Rev Food Sci Technol. (2020) 11:71–92. doi: 10.1146/annurev-food-032519-051736, PMID: 31928426

[122] Zhang ZH Liu MD Yao K Xu S Yu DX Xie DD . Vitamin D deficiency aggravates growth and metastasis of prostate cancer through promoting EMT in two β-catenin-related mechanisms. J Nutr Biochem. (2023) 111:109177. doi: 10.1016/j.jnutbio.2022.109177, PMID: 36223833

[123] Torkko K Till C Tangen CM Goodman PJ Song X Schenk JM . Vitamin D pathway and other related polymorphisms and risk of prostate cancer: results from the prostate cancer prevention trial. Cancer Prev Res (Philadelphia Pa). (2020) 13:521–30. doi: 10.1158/1940-6207.CAPR-19-0413, PMID: 32102946 PMC7272271

[124] Grimm M Cetindis M Biegner T Lehman M Munz A Teriete P . Serum vitamin D levels of patients with oral squamous cell carcinoma (OSCC) and expression of vitamin D receptor in oral precancerous lesions and OSCC. Med oral patologia Oral y cirugia bucal. (2015) 20:e188–95. doi: 10.4317/medoral.20368, PMID: 25662556 PMC4393981

[125] Chandler PD Buring JE Manson JE Giovannucci EL Moorthy MV Zhang S . Circulating vitamin D levels and risk of colorectal cancer in women. Cancer Prev Res (Philadelphia Pa). (2015) 8:675–82. doi: 10.1158/1940-6207.CAPR-14-0470, PMID: 25813525 PMC4526335

[126] Abo-Zaid MA Hamdi HA Elashmawy NF . Vitamin D and Immunity: A comprehensive review of its impact on autoimmunity, allergy suppression, antimicrobial defense, and cancer inhibition. Egyptian J Immunol. (2023) 30:47–66. doi: 10.55133/eji.300406, PMID: 37787773

[127] Lilliu H Pamphile R Chapuy MC Schulten J Arlot M Meunier PJ . Calcium-vitamin D3 supplementation is cost-effective in hip fractures prevention. Maturitas. (2003) 44:299–305. doi: 10.1016/S0378-5122(03)00038-0, PMID: 12697371

[128] Tamer G Arik S Tamer I Coksert D . Relative vitamin D insufficiency in Hashimoto's thyroiditis. Thyroid. (2011) 21:891–6. doi: 10.1089/thy.2009.0200, PMID: 21751884

[129] Muscogiuri G Mari D Prolo S Fatti LM Cantone MC Garagnani P . 25 hydroxyvitamin D deficiency and its relationship to autoimmune thyroid disease in the elderly. Int J Environ Res Public Health. (2016) 13(9):850. doi: 10.3390/ijerph13090850, PMID: 27571093 PMC5036683

[130] Yasuda T Okamoto Y Hamada N Miyashita K Takahara M Sakamoto F . Serum vitamin D levels are decreased and associated with thyroid volume in female patients with newly onset Graves' disease. Endocrine. (2012) 42:739–41. doi: 10.1007/s12020-012-9679-y, PMID: 22547366 PMC3509322

[131] Shin DY Kim KJ Kim D Hwang S Lee EJ . Low serum vitamin D is associated with anti-thyroid peroxidase antibody in autoimmune thyroiditis. Yonsei Med J. (2014) 55:476–81. doi: 10.3349/ymj.2014.55.2.476, PMID: 24532520 PMC3936621

[132] Miteva MZ Nonchev BI Orbetzova MM Stoencheva SD . Vitamin D and autoimmune thyroid diseases - a review. Folia Med (Plovdiv). (2020) 62:223–9. doi: 10.3897/folmed.62.e47794, PMID: 32666762

[133] Babic Leko M Juresko I Rozic I Pleić N Gunjača I Zemunik T . Vitamin D and the thyroid: A critical review of the current evidence. Int J Mol Sci. (2023) 24(4):3586. doi: 10.3390/ijms24043586, PMID: 36835005 PMC9964959

[134] Fang F Chai Y Wei H Wang K Tan L Zhang W . Vitamin D deficiency is associated with thyroid autoimmunity: results from an epidemiological survey in Tianjin, China. Endocrine. (2021) 73:447–54. doi: 10.1007/s12020-021-02688-z, PMID: 33759075

[135] Unal AD Tarcin O Parildar H Cigerli O Eroglu H Demirag NG . Vitamin D deficiency is related to thyroid antibodies in autoimmune thyroiditis. Cent Eur J Immunol. (2014) 39:493–7. doi: 10.5114/ceji.2014.47735, PMID: 26155169 PMC4439962

[136] Mirhosseini N Brunel L Muscogiuri G Kimball S . Physiological serum 25-hydroxyvitamin D concentrations are associated with improved thyroid function-observations from a community-based program. Endocrine. (2017) 58:563–73. doi: 10.1007/s12020-017-1450-y, PMID: 29067607 PMC5693977

[137] Aghili A Alijanpour Aghamaleki M Pornasrollah M Ghorban Nooreddini H Khafri S Alijanpour S . Effect of vitamin D therapy on hashimoto's thyroiditis in children with hypovitaminosis D. J Pediatr Perspect. (2020) 8:10889–97. doi: 10.22038/IJP.2019.42711.3579

[138] Mazokopakis EE Papadomanolaki MG Tsekouras KC Evangelopoulos AD Kotsiris DA Tzortzinis AA . Is vitamin D related to pathogenesis and treatment of Hashimoto's thyroiditis? Hell J Nucl Med. (2015) 18:222–7., PMID: 26637501

[139] Chaudhary S Dutta D Kumar M Saha S Mondal SA Kumar A . Vitamin D supplementation reduces thyroid peroxidase antibody levels in patients with autoimmune thyroid disease: An open-labeled randomized controlled trial. Indian J Endocrinol Metab. (2016) 20:391–8. doi: 10.4103/2230-8210.179997, PMID: 27186560 PMC4855971

[140] Simsek Y Cakır I Yetmis M Dizdar OS Baspinar O Gokay F . Effects of Vitamin D treatment on thyroid autoimmunity. J Res Med Sci. (2016) 21:85. doi: 10.4103/1735-1995.192501, PMID: 28163731 PMC5244647

[141] Holick MF Binkley NC Bischoff-Ferrari HA Gordon CM Hanley DA Heaney RP . Evaluation, treatment, and prevention of vitamin D deficiency: an Endocrine Society clinical practice guideline. J Clin Endocrinol Metab. (2011) 96:1911–30. doi: 10.1210/jc.2011-0385, PMID: 21646368

[142] Taheriniya S Arab A Hadi A Fadel A Askari G . Vitamin D and thyroid disorders: a systematic review and Meta-analysis of observational studies. BMC endocrine Disord. (2021) 21:171. doi: 10.1186/s12902-021-00831-5, PMID: 34425794 PMC8381493

[143] Usategui-Martın R De Luis-Roman DA Fernandez-Gomez JM Ruiz-Mambrilla M Pérez-Castrillón JL . Vitamin D receptor (VDR) gene polymorphisms modify the response to vitamin D supplementation: A systematic review and meta-analysis. Nutrients. (2022) 14(2):360. doi: 10.3390/nu14020360, PMID: 35057541 PMC8780067

